# Identification and Functional Characterisation of CRK12:CYC9, a Novel Cyclin-Dependent Kinase (CDK)-Cyclin Complex in *Trypanosoma brucei*

**DOI:** 10.1371/journal.pone.0067327

**Published:** 2013-06-21

**Authors:** Séverine Monnerat, Cristina I. Almeida Costa, Andrea C. Forkert, Corinna Benz, Alana Hamilton, Laurence Tetley, Richard Burchmore, Carlos Novo, Jeremy C. Mottram, Tansy C. Hammarton

**Affiliations:** 1 Wellcome Trust Centre for Molecular Parasitology, Institute of Infection, Immunity and Inflammation, University of Glasgow, Glasgow, United Kingdom; 2 Instituto de Higiene e Medicina Tropical, Universidade Nova de Lisboa, Lisbon, Portugal; 3 School of Life Sciences, University of Glasgow, Glasgow, United Kingdom; 4 Institute of Infection, Immunity and Inflammation, University of Glasgow, Glasgow, United Kingdom; Technion-Israel Institute of Technology Haifa 32000 Israel., Israel

## Abstract

The protozoan parasite, *Trypanosoma brucei*, is spread by the tsetse fly and causes trypanosomiasis in humans and animals. Both the life cycle and cell cycle of the parasite are complex. Trypanosomes have eleven cdc2-related kinases (CRKs) and ten cyclins, an unusually large number for a single celled organism. To date, relatively little is known about the function of many of the CRKs and cyclins, and only CRK3 has previously been shown to be cyclin-dependent *in vivo*. Here we report the identification of a previously uncharacterised CRK:cyclin complex between CRK12 and the putative transcriptional cyclin, CYC9. CRK12:CYC9 interact to form an active protein kinase complex in procyclic and bloodstream *T. brucei*. Both CRK12 and CYC9 are essential for the proliferation of bloodstream trypanosomes *in vitro*, and we show that CRK12 is also essential for survival of *T. brucei* in a mouse model, providing genetic validation of CRK12:CYC9 as a novel drug target for trypanosomiasis. Further, functional characterisation of CRK12 and CYC9 using RNA interference reveals roles for these proteins in endocytosis and cytokinesis, respectively.

## Introduction

In eukaryotes, cyclin-dependent kinases (CDKs) are of fundamental importance for cell cycle progression [1]–[3] and also play essential roles in regulating gene expression [4]–[7], autophagy [8] and neuronal function [9] as well as key roles in responding to stresses [10], [11]. CDKs are proline-directed serine-threonine kinases that are activated by the binding of a cyclin partner protein to the highly conserved PSTAIRE helix within the CDK [12], [13]. Since the cyclins regulating the cell cycle CDKs are not constitutively expressed, but instead are transcribed and degraded at specific points during the cell cycle, cyclin binding provides a cell cycle-dependent mode of CDK activation. In contrast, transcriptional cyclins are expressed at more constant levels throughout the cell cycle [14]–[16] and the neuronal CDK, CDK5, is activated by binding to the proteins p35 and p39, which do not have any sequence similarity to cyclins, but nevertheless adopt a cyclin-like fold [17]–[19]. Cyclins not only activate CDKs, but also determine the substrate specificity and/or localisation of the CDK. A CDK may bind to more than one cyclin during the cell cycle, and is thus targeted to different substrates at different phases of the cell cycle. Similarly, cyclins may bind to more than one CDK. Budding yeast express just one major cell cycle CDK, CDC28, which binds to different cyclins to promote successive cell cycle transitions [2]. On the other hand, over 20 CDKs and numerous cyclins have been identified in mammalian cells, with many able to compensate in the absence of others [1].

The protozoan parasite, *Trypanosoma brucei*, is the causative agent of African trypanosomiasis in humans and animals. Its digenetic life cycle, split between a mammalian host and the tsetse fly, is characterised by multiple differentiation events that yield a series of life cycle stages, which differ with respect to their morphology, cell structure, surface coat and biochemistry. Cell cycle control also differs between life cycle stages [20]. Only some life cycle stages, such as the procyclic form in the tsetse midgut, and the long slender trypomastigote in the mammalian bloodstream, are proliferative, while others such as the mesocyclic and metacyclic trypomastigotes in the tsetse fly and the short stumpy bloodstream form are cell cycle arrested. Eleven cdc2-related kinases (CRK1-4 and CRK6-12) and ten cyclins (CYC2-11) have been identified in *T. brucei* [20], [21]. To date, functions have only been assigned to a few of these putative regulators, as in many cases, depletion of the regulators via RNA interference (RNAi) has not resulted in a detectable phenotype [22], [23]. While this may suggest that some CRKs and cyclins are non-essential for proliferation in culture or that functional redundancy exists between them, it cannot be ruled out that the small amount of protein remaining following RNAi knockdown is sufficient for cell cycle progression. Of the CRKs with identifiable functions, CRK1 and CRK3 appear to be required for G1/S and G2/M phases of the cell cycle, respectively, in both procyclic and bloodstream trypanosomes [23], while CRK9 has been shown to phosphorylate RNA polymerase II subunit RPB1 and to be required for maturation of spliced leader RNA [24] and its depletion also causes mild defects in cell cycle progression [22]. In addition, CRK2, CRK4 and CRK6 have been postulated to play accessory roles in cell cycle progression [25]. Functions have only been discerned so far for two cyclins, CYC2/CycE1 and CYC6/CycB2, although gene knockout experiments suggest CYC3/CycB1 is also essential [26], despite RNAi experiments having yielded no detectable phenotype [27]. Depletion of CYC2 leads to an accumulation of cells in G_1_/S, and in procyclic, but not bloodstream trypanosomes, this is accompanied by microtubule extension at the posterior end of the parasite [27], [28]. Depletion of CYC6/CycB2 inhibits mitosis in procyclic and bloodstream *T. brucei*, but due to fundamental differences in cell cycle control in these life cycle stages, the downstream effects of the mitotic inhibition are quite different [27], [29]. In the absence of nuclear division, procyclic trypanosomes continue to undergo cytokinesis, while bloodstream *T. brucei* do not, but nevertheless continue to replicate DNA and organelles [29].

To date, few CRKs have been demonstrated to be cyclin-dependent in *T. brucei*. CRK3 has been shown to bind to CYC2 and CYC6 *in vivo*, and thus likely regulates G_1_/S as well as G_2_/M [26], [29]. A yeast two hybrid study identified interactions between CRK1 and CYC2/CycE1, CYC4/CycE3 and CYC5/CycE4, as well as between CRK2 and CYC2/CycE1 [30]. However, these interactions have yet to be confirmed *in vivo* in *T. brucei*, and a previous study indicated that, while CYC2 co-immunoprecipitated with CRK3, it did not interact with CRK1 or CRK2 in procyclic *T. brucei* [26]. Here we report the identification of a previously uncharacterised CRK:cyclin complex, CRK12:CYC9 in *T. brucei* and show that both CRK12 and CYC9 are essential proteins in this important pathogen.

## Materials and Methods

### Ethics statement

Animal work carried out during this study was performed under the UK Home Office Licence no. 60/3760 ‘Biochemistry, genetics and immunology of parasitic protozoa’, approved by the Animal Ethics Committee at the University of Glasgow, or under licence by the Direcção Geral de Veterinária (DGV), Portugal, according to national law no. 1005 (from 23^rd^ Oct, 1992) at the University of Lisbon. All studies were carried out by trained and licensed personnel in strict accordance with the terms of the Animal (Scientific Procedures) guidelines (1986) and the recommendations in the ‘Responsibility in the use of animals in bioscience research: Expectations of the major research council and charitable funding bodies’ document (UK) and national DGV guidelines (Portugal). Mice were euthanised before parasitaemias reached 10^9^ cells ml^−1^ by anaesthetising with carbon dioxide prior to cervical dislocation, and all other efforts were made to minimise suffering.

### Bioinformatic analyses

BLAST searches were performed via the NCBI website (http://blast.ncbi.nlm.nih.gov/Blast.cgi). Pairwise alignments were made using William Pearson’s LALIGN software available at http://www.ch.embnet.org/software/LALIGN_form.html or using VectorNTi AlignX software (Invitrogen). Cyclin (CD00043 (cyclin superfamily) and/or COG5024 (cyclin-like superfamily CCL1 (TIGR00569)) and kinase (PKc_like superfamily) domains were identified using the NCBI conserved domain search facility (http://www.ncbi.nlm.nih.gov/Structure/cdd/wrpsb.cgi) or via the website www.kinase.com. Cyclin and kinase domains were then aligned and Bootstrap Neighbour Joining trees were generated using ClustalX (1.81) [31]. HyperTree software was used to format the output of the phylogenetic trees [32].

### Culturing and transfection of trypanosomes

*T. brucei brucei* strain Lister 427 wildtype cell lines (procyclic and bloodstream stages), Lister 427 pHD449 [33] tetracycline inducible cell lines (procyclic and bloodstream stages) and the RNAi cell lines 427 pLew13 pLew29 (procyclic form), 427 pLew13 pLew90 (bloodstream stage) [34] and Lister 427 MITat1.2 clone 221a 2T1 (bloodstream stage) [35], were cultured and transfected as described previously [29], [35], [36].

### Generation of CYC9:TAP-expressing procyclic cell lines and purification of CYC9:TAP protein complexes

To create a fusion of CYC9 to the Tandem Affinity Purification tag [37] to facilitate the purification of CYC9 protein complexes, the 3′ end of the *CYC9* ORF (Tb11.01.5600) minus the stop codon (bp 451–843) was PCR-amplified using oligonucleotides OL1547 (incorporating *Nco* I and *Xho* I restriction sites) and OL1548 (with an *Nco* I restriction site) (Table S1 for all oligonucleotides used in this study), sequenced and cloned into the *Nco* I site of pGL900, in frame with the downstream *TAP* sequence. pGL900 is a derivative of the yeast C-terminal TAP tagging vector, pBS1539 (Cellzome), where a cassette consisting of *BSD^R^* flanked by tubulin intergenic sequences was cloned between the *Pst* I and *Apa* I sites, replacing the *URA3* selectable marker. The 3′ UTR of *CYC9* was then PCR-amplified with oligonucleotides OL1549 and OL1550, sequenced and cloned into the *Apa* I site of the pGL900:*CYC9:TAP* plasmid generating pGL1125. pGL1125 was linearised by digestion with *Xho* I and transfected into procyclic form *CYC9* single allele knockout (NEO^R^) parasites (see below), to replace the remaining endogenous *CYC9* allele with *CYC9:TAP*. The clones obtained were screened by PCR to confirm correct integration of *CYC9:TAP* and absence of wildtype *CYC9* alleles. The expression of CYC9:TAP was confirmed by Western blotting with anti- calmodulin binding protein (CBP; Santa Cruz) and anti-protein A (Sigma) antibodies.

CYC9:TAP protein complexes were purified from 8.8×10^10^ cells by sequential IgG and calmodulin affinity chromatography, according to [37], but adding 0.5% (rather than 0.1%) Nonidet P40 (NP40) detergent to all buffers. The success of the purification was monitored by Western blotting of appropriate samples with anti-CBP antibody. Eluates from the calmodulin column were analysed by nanoflow HPLC electrospray tandem mass spectrometry (nLC-ESI-MS/MS). 10 µl eluate were mixed with 40 µl methanol and 50 µl 25 mM ammonium bicarbonate/0.2 µgml^−1^ trypsin before being incubated at 37°C overnight. Formic acid was added to a final concentration of 1% and the sample dried via vacuum centrifugation. Tryptic peptides were solubilised in 0.5% formic acid and fractionated on a nanoflow HPLC system (Famos/Switchos/Ultimate, LC Packings) before being analysed by electrospray ionisation (ESI) mass spectrometry on a Q-STAR® Pulsar i hybrid MS/MS System. Peptide separation was performed on a Pepmap C18 reversed phase column (LC Packings), using a 5 – 85% acetonitrile gradient (in 0.5% formic acid) run over 45 min at a flow rate of 0.2 µlmin^−1^. Mass spectrometric analysis was performed using a 3 second survey MS scan followed by up to four MS/MS analyses of the most abundant peptides (3 seconds per peak) in Information Dependent Acquisition (IDA) mode, choosing 2+ to 4+ ions above a threshold of 30 counts, with dynamic exclusion for 120 seconds. Data generated were analysed using Applied Biosystems Analyst QS (v1.1) software and the automated Matrix Science Mascot Daemon server (v2.1.06) was used to interrogate *T. brucei* genome sequences. Protein identifications were assigned using the Mascot search engine, which gives each protein a probability based MOWSE score. In all cases, variable methionine oxidation was allowed in searches and carbamidomethylation of cysteines was selected as a fixed modification. Mass tolerances of 1.2 Da for MS and 0.4 Da for MS/MS analysis were used.

### Generation of cell lines expressing tyGFP:CRK12 or ty:CRK12

To facilitate immunoprecipitation of CRK12, it was tagged at its N-terminus with tyGFP as follows. The 5′ end of the *CRK12* ORF (Tb11.01.4130) was PCR-amplified from 427 genomic DNA using oligonucleotides PR32 and PR33 (introducing *Xba* I and *Xho* I restriction sites, respectively) and the 5′ end of the *CRK12* ORF was amplified using PR34 and PR35 (incorporating *Xho* I and *Bam H*I restriction sites, respectively). Both fragments were sequenced and cloned into pEnT5-GFP-TY [38] digested with *Xba* I and *Bam H*I using a threeway ligation procedure, generating pHG69, which allows expression of tyGFP:CRK12 from its endogenous locus. pHG69 was linearised by digestion with *Xho* I prior to transfection into Lister 427 wildtype and CYC9:TAP procyclic form cell lines. Expression of tyGFP:CRK12 was confirmed by Western blotting with anti-TY (BB2 antibody; [39]; a kind gift from Keith Matthews, University of Edinburgh) and anti-GFP (Santa-Cruz) antibodies.

Tetracycline inducible cell lines expressing TY-tagged CRK12 (active and kinase dead) cell lines were generated to investigate kinase activity of CRK12. The *CRK12* ORF was amplified from Lister 427 genomic DNA using PR206 and PR207 (incorporating a *TY tag* and *Bcl* I sites), cloned into pSC-B (Stratagene) and sequenced. To create a kinase dead variant of CRK12 (K358M), site directed mutagenesis was performed on the cloned *CRK12* sequence with oligonucleotides PR208 and PR209 using the QuickChange Site-Directed Mutagenesis kit (Stratagene). The *CRK12* wildtype and kinase dead sequences were then subcloned into *Bam H*I-digested pHD675 [33], generating pHG230 and pHG231, respectively. pHG230 and pHG231 were linearised by digestion with *Not* I prior to transfecting into Lister 427 pHD449 [33] cell lines. Inducible expression of the ty:CRK12 proteins was confirmed by Western blotting with anti-TY antibody (as above).

### Generation of *CYC9* knockout cell lines

Three plasmids (pGL1124, pGL1224 and pGL1217) were constructed to allow the replacement of one allele of *CYC9* with either blasticidin (*BSD^R^*), hygromycin (*HYG^R^*) or neomycin (*NEO^R^*) resistance genes, respectively. All plasmids were derived from pGL802 [40]. The *CYC9* open reading frame (ORF) 5′ and 3′ flanking regions were PCR-amplified from Lister 427 genomic DNA using the oligonucleotides OL1553 and OL1554 (5′ flank) and OL1549 and OL1550 (3′ flank), and cloned into the *Not* I/*Xba* I and *Apa* I sites of pGL802, respectively, using the restriction sites incorporated into the oligonucleotide primers, replacing the flanking regions for *MCA2* and *MCA1*, generating pGL1124. To generate pGL1224 and pGL1217, the *BSD^R^* gene was excised from pGL1124 by digestion with *Eco R*I and replaced with *HYG^R^* or *NEO^R^* genes, respectively. The *CYC9* knockout plasmids were linearised with *Xho* I prior to transfection into *T. brucei* Lister 427 cells. Trypanosomes were transfected with each plasmid individually, and then subjected to a second transfection with one of the other plasmids, with a different resistance gene, with all pairwise combinations performed.

### Generation and induction of RNAi cell lines

The RNAit software [41] was used to identify unique gene fragments suitable for RNAi studies. For *CYC9* RNAi, a 408 bp fragment of the *CYC9* ORF (bp 335–742), was PCR-amplified from Lister 427 genomic DNA using oligonucleotides OL2540 and OL2541 (incorporating *Hind* III and *Bam H*I restriction sites, respectively) and cloned into the same sites of p2T7_ti_:GFP vector [42] in place of the *GFP* coding sequence, generating pGL1759. Plasmid pGL1759 was linearised by digestion with *Not* I, transfected into the 427 pLew13 pLew29 and 427 pLew13 pLew90 RNAi cell lines, as described above and two independent clones for each cell line were selected for downstream analyses.

For RNAi of *CRK12*, a 419 bp fragment of the *CRK12* ORF (bp 1028–1446) was PCR-amplified from TREU 927 genomic DNA with oligonucleotides OL3387 (incorporating *Xma* I and *Xho* I restriction sites) and OL3388 (incorporating *Bam H*I and *Xba* I restriction sites). The product was digested with *Xho* I and *Xba* I and subcloned in an antisense orientation into the same sites in pRPa^iSL^ (MCS1/2) [35], and then digested with *Xma* I and *Bam H*I and subcloned in a sense orientation into the same plasmid, generating a stem-loop construct with a *LACZ* stuffer fragment. The resultant construct (pGL1986) was digested with *Asc* I to release the RNAi stem-loop cassette and transfected into bloodstream 2T1 cells, as described above. Hygromycin-resistant clones were analysed for puromycin sensitivity and two puromycin-sensitive clones selected for downstream analyses.

The RNAi response was induced *in vitro* by the addition of 1 µgml^−1^ tetracycline to the culture medium. Knockdown was confirmed by real time PCR, and for *CRK12* RNAi cell lines, also by Western blotting cell lysates with a specific monoclonal antibody. The CRK12 monoclonal antibody was generated by immunisation of a Balb/c mouse with purified recombinant 6xHis:CRK12 in Incomplete Freund’s Adjuvant (Sigma). Cells from the spleen were removed and fused with myeloma SP2/0 AG14 cells cultured in DMEM supplemented with 5% foetal bovine serum (Gibco) at 37°C, in the presence of 5% CO_2_, as previously described [43]. Hybridoma supernatants were screened by ELISA and Western blot to identify anti-CRK12-producing hybridomas, and clone 4D7 was selected. The antibody isotype was determined to be IgG using the Mouse MonoAB ID (alkaline phosphatase) kit (Zymed), and the antibody was used neat for Western blotting. For *in vivo* RNAi of *CRK12*, four experimental ICR mice were injected intraperitoneally with 250 µl (5×10^5^) parasites taken from a donor mouse and parasitaemias monitored daily thereafter by tail bleed. At 48 hours post-inoculation, when parasitaemias had reached >10^7^ cells ml^−1^, the drinking water of two mice was replaced with a 0.2 mgml^−1^ doxycycline/5% (w/v) sucrose solution to effect induction of the RNAi response. Mice were euthanised before parasitaemias reached 10^9^ cells ml^−1^.

### Real-time PCR analysis

Total RNA was prepared from 1×10^8^ trypanosome cells using the RNeasy Mini kit (Qiagen) and treated with 2U RNase-free DNase I (Ambion) per 10 µg RNA for 30 minutes at 37°C to digest genomic DNA before the DNase I was inactivated with 5 mM EDTA at 75°C for 10 minutes. 2 µg of RNA was used in cDNA synthesis reactions with random hexamers (Invitrogen) using the Omniscript RT kit (Qiagen), according to the manufacturer’s protocol. Real time PCR was performed as described previously [44] in triplicate (*CYC9*) or quadruplicate (*CRK12*). Dissociation curves were performed on the products to check that only one product was amplified by each primer set.

### Yeast two-hybrid analysis

To investigate the interaction between CYC9 and CRK12, the Hybrid Hunter system (Invitrogen) was used. *CYC9* was PCR-amplified from EATRO 795 genomic DNA with oligonucleotides OL1163 and OL1164 (introducing *Bam H*I and *Hind* III sites, respectively), sequenced and cloned into the same sites in the prey plasmid pYESTrp to create pGL932 (expressing B42:CYC9), while *CRK12* was amplified with OL1600 and OL1601 (incorporating *Sac* I and *Kpn* I sites, respectively), sequenced and cloned into the same sites in the bait plasmid pHybLex/Zeo, generating pGL1277 (expressing LexA:CRK12). *Saccharomyces cerevisiae* strain L40 (Invitrogen) was transformed with the two plasmids together to generate L40 pGL932 pGL1277. As autoactivation controls, the empty vector prey and bait plasmids were transformed into L40 together or in combination with pGL932 or pGL1277. Positive and negative L40 control strains, expressing LexA:Fos/B42:Jun or LexA:Lamin/B42:Jun, were also generated using plasmids supplied by Invitrogen. To analyse protein:protein interactions, β-galactosidase and histidine prototrophy assays were performed according to the manufacturer’s instructions and as previously described [45].

### Immunoprecipitation

S100 lysates were prepared from 5×10^8^ trypanosome cells as described previously [44]. Soluble cell extracts were incubated with anti-glutathione S-transferase (GST, Santa Cruz), anti-TY (BB2, [39]) or anti-rabbit IgG-conjugated Dynabeads (prepared using the Immunoprecipitation Kit - Dynabeads® Protein G, Invitrogen, according to the manufacturer’s instructions) at 4°C overnight. Beads were washed with the wash buffer supplied with the kit supplemented with 0.1% Triton X-100 and assayed for kinase activity (below) or analysed by SDS-PAGE and Western blotting using anti-GFP (Santa Cruz), anti-oligopeptidase B (OPB; [46]) and horseradish peroxidise-conjugated anti-PAP (Roche) as an anti-TAP antibody.

### Kinase assays

Kinase assays were performed as described previously [47]. Assay samples were electrophoresed on SDS-PAGE gels, which were stained with Coomassie Blue, dried and analysed by autoradiography.

### ATP assay

2×10^7^ cells were washed in Voorheis’ modified phosphate buffered saline (vPBS; [48]), resuspended in 300 µl ddH_2_O and passed through a 27G needle five times to lyse the cells. Cellular debris was removed by centrifuging at 14,000 x *g* for 5 minutes at 4°C and the supernatant was assayed for ATP activity using an ATP bioluminescent assay kit (Sigma) and an EnVision 2102 Multilabel plate reader (Perkin Elmer).

### 4,6-Diamidino-2-phenylindole (DAPI) staining and flow cytometry analysis

The nucleus/kinetoplast configurations and DNA content of cells were analyzed by DAPI staining in conjunction with fluorescence microscopy and by flow cytometry of propidium iodide stained cells, respectively, as described previously [29].

### Immunofluorescence analyses

Cells were washed in PBS (procyclic form) or vPBS (bloodstream form) and were settled onto poly-L-lysine coated glass slides (CYC9-TAP) or fixed in solution (clathrin heavy chain (CLH)). For CYC9-TAP immunofluorescence, cells were fixed in methanol at –20°C for 1 hour, before being transferred to a PBS bath for 5 minutes. Following a further PBS wash, cells were incubated with a 1/5000 dilution of rabbit anti-protein A antibody for 1 hour at room temperature. To detect CLH, cells were fixed in 3% PFA for 1 hour at 4°C before being allowed to adhere to poly-L-lysine coated slides for 20 minutes. Cells were permeabilised in PBS/0.1% Triton X-100 and neutralised in 100 mM glycine, as above, before being blocked for 1 hour in PBS/10% foetal calf serum at room temperature and then incubated with 1/500 dilution of rabbit anti-CLH antibody [49] for 1 hour at room temperature.

Following incubation in primary antibody, all cells were washed twice in PBS, and then incubated in a 1/100 dilution Alexa-Fluor 594- or Alexa-Fluor-488 conjugated anti-rabbit IgG (Invitrogen) for 1 hour in the dark. Cells were then washed twice in 100 mM 4-(2-hydroxyethyl)piperazine-1-ethanesulphonic acid (HEPES) pH 7.5, incubated with 10 µgml^−1^ DAPI in 100 mM HEPES pH 7.5 for 5 minutes, washed twice more in 100 mM HEPES pH 7.5 before SlowFade Gold antifade reagent (Invitrogen) and a coverslip were added and the cells observed using a DeltaVision RT epifluorescence imaging system (Applied Precision) and Soft WoRx software.

### Uptake assays for endocytosis

Uptake assays were performed for bloodstream form *CRK12* RNAi cell lines using the FM4-64FX (*N*-(3- triethylammoniumpropyl) -4-{6-[4-(diethylamino)phenyl]hexatrienyl} pyridinium dibromide) dye (Invitrogen) at 4°C and 37°C and AlexaFluor-594 (AF594)-conjugated human transferrin (Sigma) at 37°C essentially as described in [50], with cells being incubated with the fluorescent cargo for 5 minutes.

### Transmission Electron Microscopy

Specimens were prepared and viewed as described previously [51].

## Results

### CYC9 clusters phylogenetically with cyclin C

CYC9 is the only *T. brucei* cyclin not to have been studied to date. The *CYC9* gene comprises a 843 bp ORF that Rapid Amplification of cDNA ends (RACE) analysis revealed is flanked by a 53 nucleotide 5’ UTR and a 3’UTR of 230 or 272 nucleotides depending on which of two possible polyadenylation sites are used (data not shown). The CYC9 protein (281 amino acids) contains two cyclin-like conserved domains (IPRO11028) at residues 50-144 and 143-196 and a transcription regulation cyclin domain (IPRO15429) at residues 36-147 (www.genedb.org). A phylogenetic comparison of the cyclin domain of CYC9 with the cyclin domains of other eukaryotic cyclins reveals that it clusters tightly with CYC9 from *T. cruzi* and *Leishmania* species, and clusters more closely with transcriptional cyclins (human and *Drosophila* cyclins C, H, K, L and T, as well as with fission yeast Srb11, a cyclin C orthologue) than any other class of cyclin (Fig. S1A).

### CYC9 associates with CRK12 in vivo

**Figure 1:**
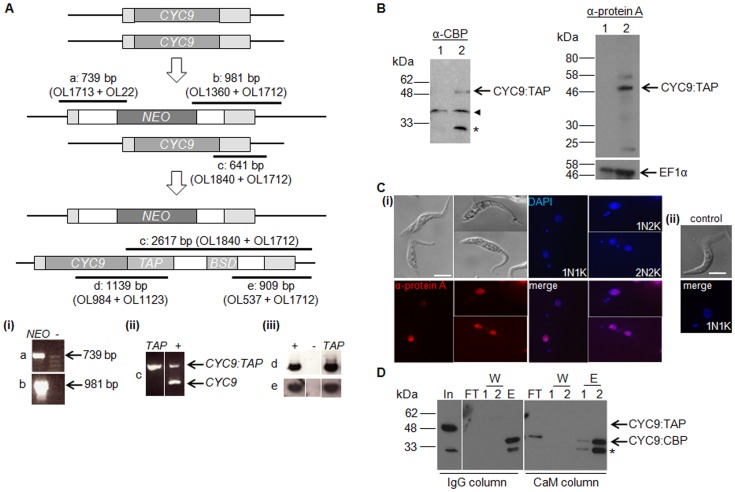
Identification of CRK12 as a CYC9 interaction partner using tandem affinity purification. A. Construction of procyclic CYC9:TAP cell line. *CYC9* alleles were replaced sequentially with a neomycin resistance (*NEO*) cassette and a *CYC9:TAP* cassette, which included a blasticidin resistance marker (*BSD*) for selection, thus generating a cell line expressing CYC9:TAP in a *CYC9* null mutant background. Light grey boxes: *CYC9* UTR sequences; white boxes: tubulin intergenic sequences. PCR (reactions a-e, sizes and positions of products amplified indicated by black bars) was used to verify correct integration of the cassettes. Reactions (a) and (b) (panel (i)), were used to confirm correct integration of the *NEO* cassette in the single allele *CYC9* knockout line; negative control (–): procyclic 427 wildtype. Reaction (c) (panel (ii)) was used to confirm that a wildtype copy of *CYC9* no longer existed in the *CYC9:TAP* cell line; positive control (+): procyclic 427 wildtype cell line transfected with *CYC9:TAP* cassette. Reactions (d) and (e) (panel (iii)), were used to confirm correct integration of the *CYC9:TAP* cassette at its 5’ and 3’ ends; positive and negative controls as for (c) and (b), respectively. B. Western blots probed with anti-CBP (left), anti-protein A (right, upper blot) and EF1α (loading control; right, lower blot) antibodies. Lane 1: procyclic 427 transfected with *NEO* cassette; lane 2: as for lane 1 but also transfected with CYC9:TAP construct. Expected size of CYC9:TAP is 51.6 kDa. Asterisk: degradation product; arrowhead: cross-reacting band that serves as a loading control. C. (i) Immunofluorescence analysis of procyclic CYC9:TAP cell line. Top left: DIC; top right: DAPI; bottom left: anti-protein A (CYC9:TAP); bottom right: DAPI/anti-protein A merge. (ii) Wildtype control. Top: DIC; bottom: DAPI/anti-protein A merge. The configuration of nuclei (N) and kinetoplasts (K) per cell is given. Scale bar: 5 µm. D. Western blot of CYC9:TAP purification, probed with anti-CBP. In: input to the IgG column; FT: flow through; W: washes; E: elution fractions. Expected size of CYC9:CBP (after cleavage of CYC9:TAP from the IgG column with TEV protease) is 35.5 kDa. Asterisk: degradation product.

To enable functional characterisation of CYC9, CYC9 was tagged at its C-terminus with a Tandem Affinity Purification (TAP) [37] tag (CYC9:TAP) in procyclic *T. brucei* to facilitate the identification of *in vivo* kinase partner(s) of CYC9. A procyclic cell line was generated where one *CYC9* allele was replaced with *CYC9:TAP* and the other allele was replaced by a neomycin resistance gene, and PCR was used to confirm correct integration (Fig. 1A). Hence, *CYC9:TAP* was the only expressed copy of *CYC9* in this cell line, and since data described below indicate that *CYC9* is an essential gene, this points to CYC9:TAP being functional. Expression of CYC9:TAP (51.6 kDa) was confirmed by Western blotting with anti-Calmodulin Binding Protein (CBP) and anti-protein A antisera (Fig. 1B) and by immunofluorescence analysis with the anti-protein A antibody, which showed CYC9:TAP to be localised to the nucleus (Fig. 1C). CYC9:TAP was expressed in cells of all cell cycle stages, as expected due to the tubulin 3′ UTR sequence downstream of the tagged gene. CYC9:TAP complexes were purified via affinity purification, firstly on IgG sepharose. Following binding of the CYC9:TAP complexes to the beads and extensive washing, CYC9 complexes were released from the beads by TEV protease cleavage. The resultant CYC9:CBP (35.5 kDa) and associated protein(s) were then bound to calmodulin resin, washed and eluted by the addition of EGTA. The success of the purification was assessed by Western blotting with anti-CBP (Fig. 1D). In the cell lysate, two protein species were recognised by the antibody, one at ∼50 kDa, consistent with it being CYC9:TAP, and a smaller one of ∼30 kDa, which most likely represented a degradation product of CYC9:TAP since it was also present in the elutions from the second affinity chromatography step. Following TEV protease treatment, a ∼35 kDa protein was detected, most likely CYC9:CBP, indicating that CYC9:CBP and associated protein(s) were successfully purified by tandem affinity purification. Mass spectrometry of elution 3 identified two peptides, KMDAIDEVVRL and KKLHEMGIIHRD, that corresponded to *T. brucei* CRK12 (amino acids 404-414 and 464-475, respectively), strongly suggesting that CYC9 binds to CRK12 *in vivo*.

To confirm the interaction between CYC9 and CRK12, two other approaches were taken. Firstly, a yeast two-hybrid assay was used. The *CRK12* and *CYC9* open reading frames were cloned into the bait (pHybLex/Zeo) and prey (pYESTrp) vectors, respectively, such that CRK12 was expressed as a LexA binding domain fusion (LexA:CRK12; pGL1277) and CYC9 was expressed fused to the B42 activating domain (B42:CYC9; pGL932), and the plasmids were transformed into the yeast-two hybrid reporter strain L40. L40 co-transformed with both pGL1277 and pGL932 plasmids exhibited β-galactosidase activity (Fig. S2A), indicating an interaction between LexA:CRK12 and B42:CYC9, since neither LexA:CRK12 or B42:CYC9 alone was able to induce expression of *LacZ*. Additionally, yeast expressing LexA:CRK12 and B42:CYC9 displayed histidine autotrophy (Fig. S2B), providing further evidence that these proteins interact in yeast.

**Figure 2:**
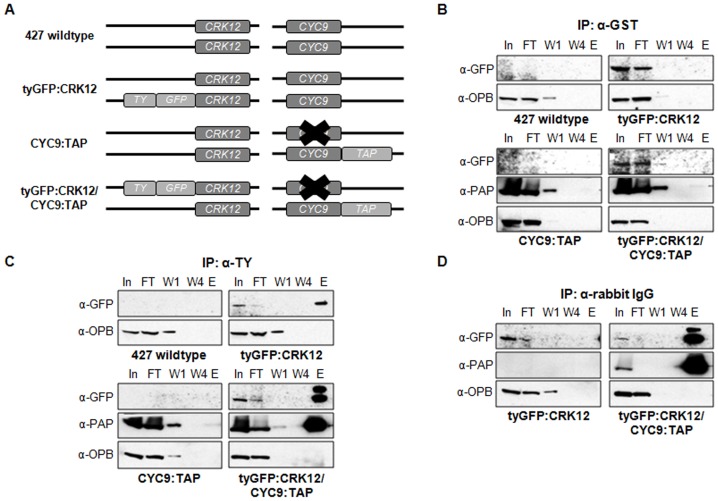
Co-immunoprecipitation of CRK12 and CYC9. A. Schematic showing features of procyclic cell lines generated. B–D. Immunoprecipitation (IP) was performed with (B) an irrelevant antibody (anti-GST), (C) anti-TY antibody (for tyGFP:CRK12) and (D) anti-rabbit IgG (for CYC9:TAP). Samples (In: input; FT: flow through; W1: first wash; W4: last wash; E: elution) were analysed by Western blotting with anti-GFP to detect tyGFP:CRK12, anti-PAP to detect CYC9:TAP and anti-oligopeptidase B (anti-OPB) to act as a loading control for the input fractions and control for the stringency of the purification.

Secondly, co-immunoprecipitation of CRK12 and CYC9 from *T. brucei* cell lysates was demonstrated (Fig. 2 and S3). CRK12 was tagged at its N-terminus with TY1 [39] and GFP epitopes (tyGFP:CRK12) to facilitate its detection, and expressed from the *CRK12* endogenous locus in either a wildtype or a CYC9:TAP background (Fig. 2A). Use of tagged CYC9 was required since useful anti-CYC9 antibodies could not be generated. Immunoprecipitations were performed by incubating cell lysates of these cell lines with beads conjugated to anti-TY antibody (to precipitate tyGFP:CRK12), anti-rabbit IgG (to precipitate CYC9:TAP) or anti-GST antibody (a specificity control) and appropriate samples analysed by Western blotting with anti-GFP (to detect tyGFP:CRK12), anti-PAP (to detect CYC9:TAP) and anti-OPB or anti-EF1α (loading control) antibodies (Fig. 2). Anti-GST beads did not pull down tyGFP:CRK12 or CYC9:TAP from cell lysates, demonstrating that neither of these proteins bound to the beads non-specifically (Fig. 2B). Anti-TY beads immunoprecipitated tyGFP:CRK12 and CYC9:TAP was coprecipitated (Fig. 2C). Similarly, anti-rabbit IgG beads immunoprecipitated CYC9:TAP and co-precipitated tyGFP:CRK12 (Fig. 2D), thus confirming that CRK12 and CYC9 interact *in vivo* in procyclic *T. brucei*. Tagged cell lines were also generated in the bloodstream form; CYC9:TAP was found to interact with tyGFP:CRK12 (Fig. S3A) and was localised in the nucleus (Fig. S3B) as in the procyclic form. Bioinformatic searches did not reveal the presence of a conventional nucleus localisation signal (NLS) in CYC9 (data not shown), but this does not preclude there being one, since NLS consensus sequences are not well defined in *T. brucei*.

### CRK12 autophosphorylates in vivo

**Figure 3:**
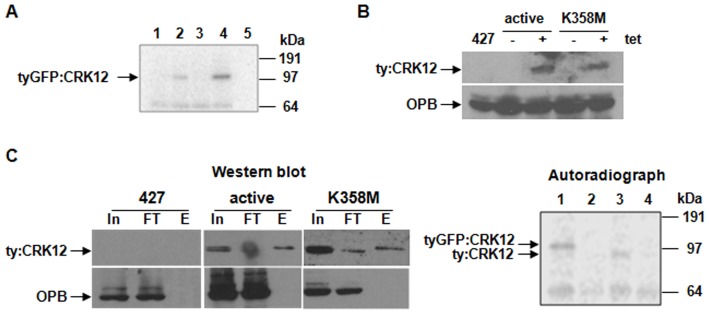
CRK12 is an active protein kinase. A. Anti-TY immunoprecipitates from procyclic cell lysates (1: 427 wildtype; 2: 427 tyGFP:CRK12; 3: 427 CYC9:TAP; 4: 427 tyGFP:CRK12 CYC9:TAP, see Fig. 2A) were subjected to a radiolabelled *in vitro* kinase assay and analysed by SDS-PAGE and autoradiography. Lane 5: kinase assay buffer only. Bands likely representing phosphorylated tyGFP:CRK12 (113.5 kDa) are indicated. B: Western blot of 427 cell lysates overexpressing ty:CRK12 (active) or kinase dead ty:CRK12 (K358M) in response to tetracycline (tet) induction. A 427 wildtype cell lysate is included as a negative control. Western blots were probed with anti-TY antibody to detect ty:CRK12 or anti-OPB as a loading control, as indicated. C. Immunoprecipitation of ty:CRK12 performed using anti-TY beads and 427 cell lysates overexpressing either ty:CRK12 (active) or kinase dead ty:CRK12 (K358M). 427 wildtype lysates were included as a non-specific binding control. Western blots (left) were performed on the input (In), flow through (FT) and elution (E) fractions with anti-TY antibody to detect ty:CRK12 or with anti-OPB antibody as a control for non-specific binding to the beads, as indicated. Immunoprecipitates were then subjected to a radiolabelled kinase assay (autoradiograph, right). 1: 427 tyGFP:CRK12; 2: 427 wt; 3: 427 ty:CRK12 (active); 4: 427 ty:CRK12 K358M. Bands likely representing phosphorylation of tyGFP:CRK12 (113.5 kDa) or ty:CRK12 (86 kDa) are indicated.

To determine whether CRK12 is an active protein kinase, lysates of the cell lines described above (Fig. 2A) were incubated with anti-TY beads; the beads were then washed extensively and used in *in vitro* kinase assays (Fig. 3). No specific protein kinase activity against several generic substrates (histone H1, myelin basic protein, alpha and beta casein) was observed (not shown), but a protein, consistent in size with tyGFP:CRK12, and only present in assays using cell lysates expressing tyGFP:CRK12 was phosphorylated (Fig. 3A), indicating that either tyGFP:CRK12 is able to autophosphorylate, or that a protein kinase(s) non-specifically interacting with the beads was/were able to phosphorylate it.

In order to distinguish between these possibilities, and to rule out that the observed phosphorylation was occurring on the GFP tag rather than on CRK12, two new cell lines were generated that inducibly expressed ty:CRK12, either wildtype (kinase active) or with a mutation (K358M) of the invariant catalytic lysine residue of the protein kinase domain predicted to result in a dead kinase. Both ty:CRK12 and ty:CRK12 (K358M) were inducibly expressed at similar levels following the addition of tetracycline (Fig. 3B). Immunoprecipitated wildtype ty:CRK12 was phosphorylated while ty:CRK12 (K358M) was not, indicating that the phosphorylation occurred as a result of autophosphorylation by the active kinase (Fig. 3C).

### CYC9 is essential in procyclic and bloodstream stage *T. brucei*

To investigate the function of CYC9, attempts were made to knockout the *CYC9* gene in procyclic and bloodstream trypanosomes. First, one allele of the gene was replaced with either a blasticidin, neomycin or hygromycin resistance marker. Single allele *CYC9* knockout mutants were obtained for both life cycle stages (Fig. S4) and were then transfected with a different resistance construct to try to delete the second allele. This was unsuccessful in all cases; either no clones were obtained from the transfection (despite multiple attempts) or double drug resistant clones were subsequently found to still have a copy of *CYC9* present, with the second drug resistance marker having integrated erroneously elsewhere in the genome (Fig. S4). Attempts to generate a conditional *CYC9* null mutant, where an ectopic copy of *CYC9* (ha:CYC9) under tetracycline-inducible control was introduced prior to knocking out the second allele, also failed. Overexpression of ha:CYC9 was not stable, with expression of ha:CYC9 falling to undetectable levels within a few days, suggesting that overexpression of ha:CYC9 was toxic.

**Figure 4:**
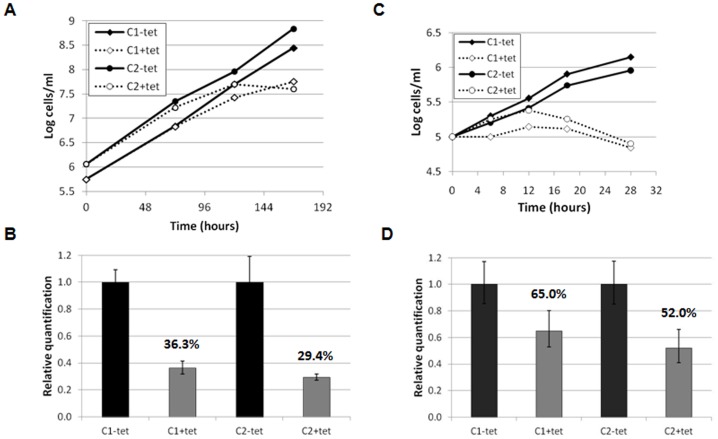
RNAi of *CYC9* reveals it is essential for viability in procyclic and bloodstream form trypanosomes. A. Cumulative growth curves for two independent procyclic *CYC9* RNAi clones (clone 1: C1; clone 2: C2) cultured in the presence or absence of tetracycline (tet). B. Real time PCR analysis of *CYC9* transcript for procyclic *CYC9* RNAi clones 1 and 2 at 48 hours post-induction. Error bars represent standard deviations of three replicates. The percentage of mRNA transcript remaining is indicated. C. Cumulative growth curves for two independent bloodstream *CYC9* RNAi clones (clone 1: C1; clone 2: C2) cultured in the presence or absence of tetracycline (tet). D. Real time PCR analysis of *CYC9* transcript for bloodstream form *CYC9* RNAi clones 1 and 2 at 20 hours post-induction. Error bars represent standard deviations of three replicates.

An RNAi approach was therefore taken to investigate CYC9 function. Tetracycline-inducible *CYC9* RNAi procyclic and bloodstream cell lines were generated, and two independent clones of each life cycle stage were selected for downstream analyses. Induction of *CYC9* RNAi in procyclic *T. brucei* resulted in a decreased growth rate or growth arrest from 120 hours post-induction (Fig. 4A). Real time PCR demonstrated that *CYC9* mRNA was significantly depleted from induced cells at 48 hours post-induction (Fig. 4B). Induction of *CYC9* RNAi in bloodstream stage *T. brucei* also lead to a growth arrest, which was apparent from 12–18 hours post-induction (Fig. 4C). Real time PCR indicated that *CYC9* mRNA was reduced by 35–50% at 20 hours post-induction (Fig. 4D). Thus, CYC9 is essential for proliferation for both procyclic and bloodstream stage *T. brucei*.

### CYC9 depletion does not result in a cell cycle block in procyclic *T. brucei*

To determine whether the growth arrest observed following knockdown of *CYC9* occurred as the result of a cell cycle arrest, RNAi cells were examined by DAPI staining to determine the nucleus/kinetoplast (N/K) configurations of cells and by flow cytometry to measure DNA content. RNAi of *CYC9* in procyclic cells resulted in only minor changes to N/K configurations (Fig. S5A). The proportion of 1N1K cells increased slightly, while the proportions of 1N2K and 2N2K cells decreased slightly during the induction period, indicating a slight accumulation of cells (5–10% extra 1N1K cells) in G_1_ phase of the cell cycle. This effect was more obvious for clone 2, in which CYC9 was knocked down to a greater extent (Fig. 4B). However, depletion of CYC9 did not result in an efficient G_1_ cell cycle block, and abnormal cells including zoids (0N1K), 1N0K and 2N1K also arose in small numbers following depletion of CYC9. Flow cytometry profiles (Fig. S5B) were consistent with the DAPI staining; a slight increase in the 2C peak was observed following induction for clone 2, and a small <1C peak appeared at later time points, most likely reflecting the generation of zoids. Thus, although CYC9 is essential for *in vitro* growth of *T. brucei*, it does not appear to play a key role in cell cycle progression in procyclic trypanosomes.

### Depletion of CYC9 blocks cytokinesis in bloodstream stage *T. brucei*

**Figure 5:**
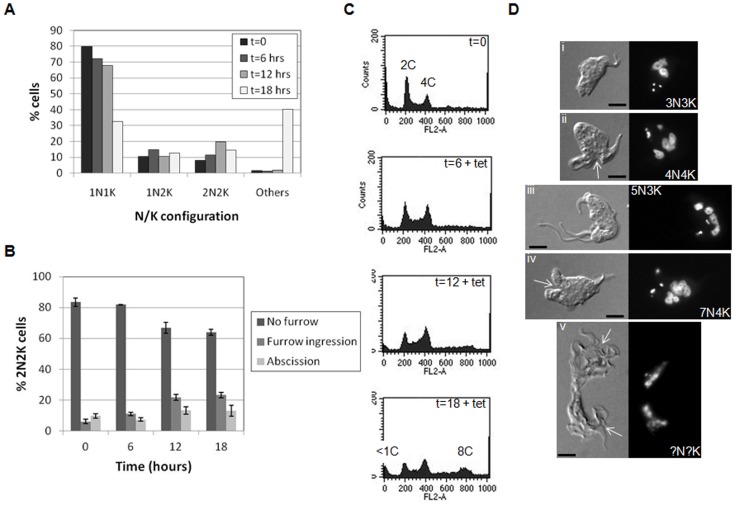
Depletion of *CYC9* in the bloodstream form inhibits cytokinesis. A. DAPI staining of nuclei (N) and kinetoplasts (K) at the time points indicated (post-induction). >200 cells per time point were classified according to their N/K configuration. B. Cytokinesis stage analysis of 2N2K cells. 2N2K cells (*n* > 200/timepoint) were scored by differential interference contrast (DIC) microscopy for whether or not they had a visible cleavage furrow (furrow ingression) or were undergoing abscission. Error bars represent the standard deviations from three replicate experiments. C. Flow cytometry analysis of propidium iodide stained cells at the time points indicated (in hours). The ploidies of the peaks are indicated. D. Example images of multinucleate/kinetoplast cells. Left panels: DIC image; right panels: DAPI staining. The N/K configuration of each cell is indicated. Scale bars: 5 µm. Arrows indicate partially ingressed cleavage furrows.

In contrast to depletion of CYC9 in procyclic trypanosomes, RNAi of *CYC9* in bloodstream parasites resulted in aberrant cell cycle progression. Since data for the two independent clones were very similar, only data for one clone is presented here. DAPI staining revealed a two fold increase in 2N2K cells over the first 12 hours of induction (Fig. 5A). Examination of 2N2K cells revealed that while the majority of this cell type had not yet commenced cytokinesis, over time, the proportion of 2N2K cells with a visible cleavage furrow significantly increased (from 6%–23% (one way ANOVA; F = 176.58, df = 5, p = 0.00); Fig. 5B). Taken together, these data suggest that depletion of CYC9 inhibits cytokinesis. At 18 hours post-induction, a population of cells with abnormal N/K configurations (many of which contained multiple nuclei and kinetoplasts) and increased ploidy was detected by DAPI staining (‘others’ in Fig. 5A) and flow cytometry (Fig. 5C; 8C peak), respectively. While some of these multi-nucleate cells did not appear to have commenced cytokinesis (Fig. 5Di and iii), others were observed to have an invagination at their anterior end (Fig. 5Dii and iv), suggesting they had attempted to begin furrow ingression, and some were observed to be attempting to undergo abscission, although since some cells were observed to have multiple cell bodies still attached at their posterior ends (Fig. 5Dv), it is likely that completion of cytokinesis was inhibited (Fig. 5D).

### CRK12 displays similarity to PITSLRE protein kinases, but forms a separate phylogenetic clade

CRK12 contains a PITSLRE motif in its PSTAIRE box, characteristic of metazoan CDK11 [52]. BLAST searches were performed for CRK12 revealing that it did indeed display similarity to other PITSLRE kinases, but was also similar to the transcriptional CDKs, CDK9 and CDK12 [52] (data not shown). However, a bootstrapped phylogenetic tree of all of the *T. brucei* CRKs, other kinetoplastid CRK12 kinases, all 21 human CDKs and selected CDKs from *Drosophila melanogaster* and *Caenorrhabditis elegans* revealed that the kinetoplastid CRK12 proteins formed a separate clade and were more similar to *T. brucei* CRK8 and CRK11 than to the PITSLRE clade (Fig. S1B). Thus CRK12 might have novel functions in *T. brucei*.

### CRK12 is an essential protein in bloodstream stage *T. brucei*

**Figure 6:**
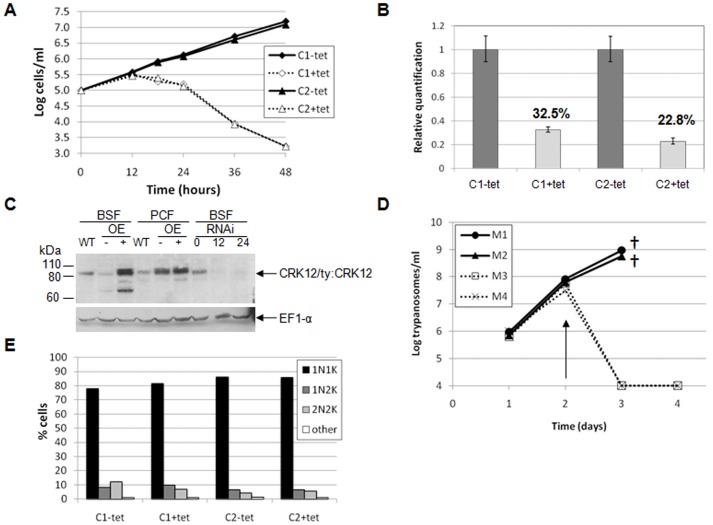
*CRK12* is essential for viability in the bloodstream form. A. Cumulative growth curves for two *CRK12* RNAi clones (C1 and C2) cultured in the presence or absence of tetracycline (tet). B. Real time PCR analysis of *CRK12* transcript at 18 hours post-induction (% mRNA transcript remaining is indicated). Error bars: standard deviations of four replicates. C. Western blot analysis of CRK12 protein depletion following RNAi induction. Cell lysates (10^6^ cell equivalents/lane) of *CRK12* RNAi clone 1 at 0, 12 and 24 hours post-induction were analysed by Western blotting with anti-CRK12 antibody (upper blot). CRK12/ty:CRK12 (85/86 kDa) is indicated. To demonstrate anti-CRK12 monoclonal antibody specificity, procyclic (PCF) and bloodstream form (BSF) cell lysates of 427 wildtype (WT) and 427 pHD449 pHG230 (ty:CRK12 inducible overexpression cell line, OE, induced (+) or not (–) with tetracycline for 24 (bloodstream form) or 48 (procyclic form) hours) were also blotted. As a loading control, blots were probed with anti-EF1α antibody (lower blot). D. Growth curves of *CRK12* RNAi cell lines in a mouse model. Mice were inoculated intraperitoneally with 5×10^5^ parasites on day 0; mice 3 and 4 were provided with doxycycline in their drinking water at day 2 (indicated with arrow) to induce the RNAi. Mice 1 and 2 were euthanised on day 3 due to their high parasitaemias. E. DAPI staining of nuclei (N) and kinetoplasts (K) at the time points indicated for *CRK12* RNAi clones 1 and 2 induced or not with tetracycline (tet). >200 cells per time point were classified according to their N/K configuration.

To investigate the function of CRK12, RNAi was used to deplete CRK12 from *T. brucei*. Previously, downregulation of CRK12 from procyclic *T. brucei* did not lead to a discernible phenotype [22], and hence our work focussed on the bloodstream stage. Tetracycline-induction of two independent *CRK12* bloodstream stage RNAi cell lines arrested growth within 18 hours, and cells died following longer exposure to tetracycline (Fig. 6A). *CRK12* mRNA was depleted to around 30% of uninduced levels at 18 hours post-induction (Fig. 6B) and Western blotting with an anti-CRK12 monoclonal antibody demonstrated that CRK12 protein was also substantially depleted by 12 hours after RNAi induction (Fig. 6C). Western blotting cell extracts from procyclic and bloodstream cell lines overexpressing ty:CRK12 confirmed the specificity of the antibody. However, all attempts to detect CRK12 by immunofluorescence have been unsuccessful to date. The importance of CRK12 for proliferation of bloodstream *T. brucei* in a mouse model was also investigated. Mice were inoculated with *CRK12* RNAi trypanosomes and the RNAi response induced via doxycycline added to their drinking water. Non-doxycycline treated mice had to be culled at 72 hours post-infection due to their high parasitaemias, while doxycycline-treated mice had no detectable parasitaemia at 72 or 96 hours post-infection (Fig. 6D), indicating that CRK12 is essential for survival of *T. brucei* in mice.

### Depletion of CRK12 results in enlargement of the flagellar pocket and defects in endocytosis

**Figure 7:**
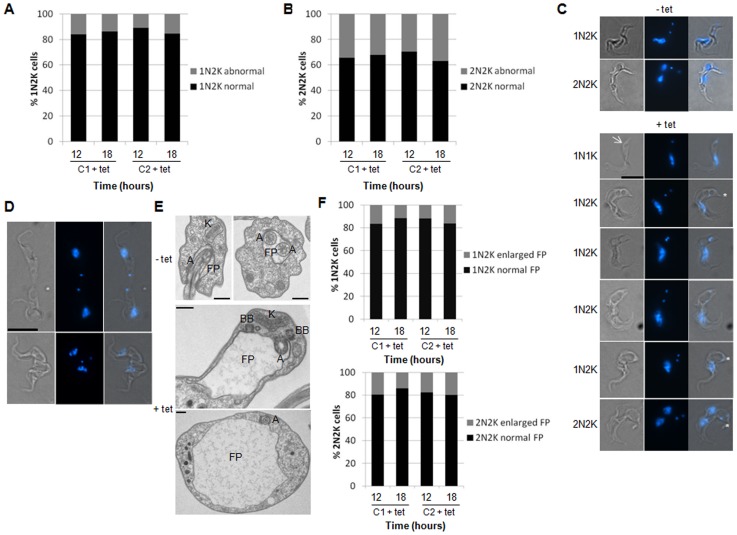
Depletion of CRK12 in bloodstream form *T. brucei* results in a defect in endocytosis. A and B. Quantification of 1N2K and 2N2K cells, respectively, with normal and abnormal kinetoplast positioning at 12 and 18 hours post-induction with tetracycline (tet). No cells were observed to have abnormal kinetoplast positioning at 0 hours. *n* >200 cells/time point. C. Visualising enlarged flagellar pockets and mispositioned kinetoplasts. Uninduced cells are shown at the top for comparison. Example images of induced cells with abnormally enlarged flagellar pocket regions (one example indicated by arrow) are shown below. From left to right: DIC image, DAPI image, DIC/DAPI merge. The N/K configuration of each cell is indicated. Note the lateral positioning of the 2 kinetoplasts in some induced cells (indicated by asterisks) compared to the longitudinal positioning in the uninduced cells. Scale bar: 10 µm. D. Example images of cells in abscission with enlarged flagellar pocket regions. From left to right: DIC image, DAPI image, DIC/DAPI merge. Scale bar: 10 µm. E. Transmission electron microscopy (TEM) images of flagellar pockets in *CRK12* RNAi cells induced (+) or not (–) with tetracycline (tet) (t = 18 hrs). A: axoneme; BB: basal body; FP: flagellar pocket; K: kinetoplast. Scale bars: 500 nm. F. Quantification of 1N2K (top) and 2N2K (bottom) cells with normal and enlarged flagellar pockets (FP) at 12 and 18 hours post-induction with tetracycline (tet). No cells were observed to have enlarged flagellar pockets at 0 hours. *n* >200 cells/time point.

Cell cycle progression was examined in *CRK12* RNAi cell lines cultured *in vitro*. DAPI staining (Fig. 6E) and flow cytometry (data not shown) did not reveal any major defects in cell cycle progression. However, closer examination revealed defects in kinetoplast positioning in 1N2K and 2N2K cells at 12 and 18 hours post-induction (Fig. 7A,B), which were not observed in uninduced cells. Instead of the kinetoplasts being arranged longitudinally along the cell axis, 1N2K and 2N2K cells with the two kinetoplasts positioned laterally across the cell accumulated (Fig. 7A–C). Many of these cells also had enlarged flagellar pockets (Fig. 7C–F), which could be visualised by DIC microscopy (Fig. 7C,D) and by TEM (Fig. 7E), that might have caused the distortion in kinetoplast position. 1N1K cells with an enlarged flagellar pocket were also observed occasionally (Fig. 7C), but the flagellar pocket enlargement was not as substantial as in 1N2K and 2N2K cells, suggesting that flagellar pocket enlargement increased as the cell progressed through the cell cycle. The majority of 2N2K cells were able to undergo cytokinesis regardless of whether they possessed enlarged flagellar pockets, although defects in cytokinesis were observed in some cells (Fig. 7D).

**Figure 8:**
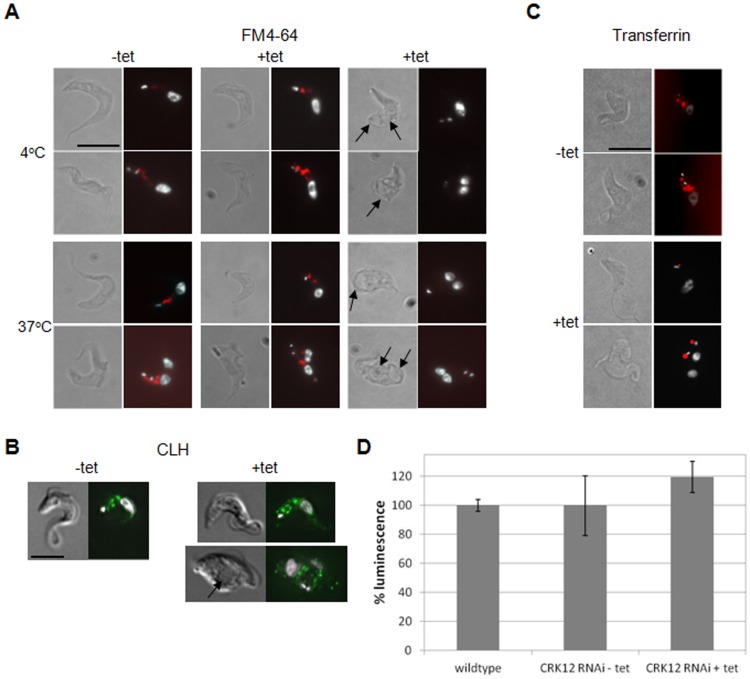
CRK12 depleted bloodstream form *T.brucei * exhibit defective FM4-64 uptake and receptor-linked endocytosis. A. FM4-64 uptake assay at 4°C and 37°C for *CKR12* RNAi cells (clone 1) induced or not with tetracycline (tet) for 18 hours. For each pair of images: left: DIC images; right: DAPI (white)/FM4-64 (red) merge. Two sets of + tet images are shown: those without enlarged flagellar pockets (centre panels) and those with enlarged flagellar pockets (right panels, as indicated by arrows). B. Clathrin heavy chain (CHC) immunofluorescence analysis of *CRK12* RNAi cells (clone 1) induced or not with tetracycline (tet) for 12 hours. Left: DIC images; right: DAPI (white)/CHC (green). Induced cells exhibiting normal (upper panels) and enlarged (lower panels, as indicated by arrow) flagellar pockets are shown. C. AF594-transferrin uptake assay at 37°C for *CKR12* RNAi cells (clone 1) induced or not with tetracycline (tet) for 18 hours. Left: DIC images; right: DAPI (white)/AF594-transferrin (red) merge. Scale bars: 10 µm. D. Bioluminescent intracellular ATP assay for 427 wildtype and *CRK12* RNAi cells (induced or not with tetracycline (tet)). Assays were performed in quadruplicate and the luminescence obtained was averaged and normalised to the wildtype control. Error bars show the standard deviations.

An enlarged flagellar pocket has previously been linked to defects in endocytosis [53]. To determine whether CRK12-depleted cells with enlarged flagellar pockets had defects in endocytosis, uptake of the fluorescent lipophilic dye, FM4-64, was analysed. While FM4-64 was efficiently taken up and internalised by uninduced cells at 4°C and 37°C (Fig. 8A, left panels), as well as by induced *CRK12* RNAi cells with no obvious enlargement to their flagellar pocket (Fig. 8A, middle panels), induced cells with enlarged flagellar pockets showed no FM4-64 signal under identical conditions (Fig. 8A, right panels). We therefore hypothesised that cells with significant flagellar pocket defects were unable to internalise the dye due to blocked endocytosis, and that the dye then diffused out of the flagellar pocket when cells were washed prior to imaging. The trypanosome clathrin heavy chain (CLH) is required for endocytosis at the flagellar pocket, and localises to numerous tubule-vesicular structures in the cytoplasm [49]. Immunofluorescence with an anti-CLH antibody revealed a normal distribution of CLH in uninduced and induced *CRK12* RNAi cells without enlarged flagellar pockets, but in *CRK12* RNAi cells with enlarged flagellar pockets, clathrin was not internalised and instead was restricted to the periphery of the flagellar pocket (Fig. 8B), thus corroborating the FM4-64 data that endocytosis is inhibited in induced *CRK12* RNAi cells with enlarged flagellar pockets. Uptake of AF594-transferrin at 37°C was also examined in *CRK12* RNAi cells. In contrast to FM4-64, AF 594-transferrin, which is taken up by receptor-linked endocytosis [54], was not internalised by induced *CRK12* RNAi cells and remained in the flagellar pocket, regardless of pocket size (Fig. 8C). Thus it appears that depletion of CRK12 blocks receptor-linked endocytosis (AF594-transferrin), and also blocks endocytosis of FM4-64 in some cells. Since endocytosis in BSF parasites is energetically expensive, ATP levels were assayed to determine whether *CRK12* depletion resulted in a reduction in intracellular ATP concentration that might account for the observed defects in endocytosis. However, ATP levels in induced *CRK12* RNAi cells were found to be equivalent to those in uninduced and wildtype 427 cells (Fig. 8D).

## Discussion

We report the identification and characterisation of the third CRK:cyclin complex *in vivo* in *T. brucei*. CRK12:CYC9 interact in a yeast two-hybrid assay and form an active protein kinase complex in procyclic and bloodstream form *T. brucei*. Intriguingly, although CRK12 was able to autophosphorylate, it was unable to phosphorylate several common generic CDK substrates, suggesting that it may have very narrow substrate specificity. Given that this is the first CDK to be linked to a role in endocytosis (see below), it may perform this function by phosphorylating a trypanosome-specific substrate.

RNAi depletion experiments indicate that both partner proteins are essential; CYC9 was essential for proliferation in culture of both procyclic and bloodstream trypanosomes, while CRK12 was found to be essential for proliferation of bloodstream trypanosomes *in vitro* and *in vivo* in mice. Our CRK12 data is in agreement with another study published recently, which identified CRK12 as an essential protein kinase in bloodstream form *T. brucei* [*55*]. In contrast, CRK12 may not be required for the proliferation of procyclic *T. brucei* [22], which given the apparent role of CRK12 in endocytosis (see below), might reflect the downregulation of endocytosis in this life cycle stage [56], as has been hypothesised to account for stage-specific differences in sensitivity to depletion of the late endosomal protein, RAB7 [57]. Our data provide genetic validation of CRK12:CYC9 as a potential novel drug target for African trypanosomiasis and future work should focus on identifying substrates to allow the development of an *in vitro* assay for this kinase complex that would facilitate high throughput screening for small molecule inhibitors.

This study began the functional characterisation of CRK12:CYC9. CYC9 most closely clusters phylogenetically with transcriptional cyclins. CRK12 has a ‘PITSLRE’ motif in its protein kinase domain located towards the C-terminus of the protein [20] and thus resembles PITSLRE kinases including metazoan CDK11 [58]. There are many isoforms of CDK11 [59], [60], some of which regulate splicing and transcription [58], [61]–[63] while others are required for centrosome maturation, mitotic spindle formation and sister chromatid cohesion [64], [65], or are involved in cytokinesis [66]–[69]. BLAST analyses also revealed similarity between CRK12 and the transcriptional kinases CDK9 and CDK12. However, phylogenetic analysis shows that the trypanosomatid CRK12 proteins form their own clade separate from the PITSLRE and transcriptional CDK clades, and thus may have evolved their own novel functions. Indeed, depletion of CRK12 from bloodstream stage *T. brucei* resulted in flagellar pocket enlargement, suggestive of a defect in endocytosis [53]. Our data showing a lack of AF594-transferrin internalisation, reduction in FM4-64 uptake and mislocalisation of clathrin heavy chain following CRK12 depletion supports CRK12 playing a critical role in endocytosis, a function which to our knowledge, has not been described for any CDK previously. CRK12 could directly regulate endocytosis, by phosphorylating a component of the endocytic machinery, or could do so indirectly by phosphorylating a nuclear factor that regulates the expression of genes involved in endocytosis. Future work should focus on determining the localisation of CRK12 to help shed further light on its function. We could not, however, detect a role for CRK12 in regulating the *T. brucei* cell cycle.

Depletion of CYC9 gave rise to different phenotypes in bloodstream and procyclic life cycle stages, which could be due to CYC9 interacting with additional different CRKs in the different life cycle stages, or because CRK12:CYC9 phosphorylates different substrates according to the life cycle stage. In bloodstream stage *T. brucei*, depletion of CYC9 rapidly led to a two fold increase in 2N2K cells with an increased proportion undergoing furrow ingression, and later, these cells continued to re-replicate their organelles despite not having completed division, and even attempted to divide again, resulting in cells with multiple cell bodies. Given its nuclear localisation, and its similarity to transcriptional cyclins, it is unlikely that CYC9 directly effects cytokinesis, but it could regulate gene expression of cytokinesis effectors which are not expressed constitutively throughout the cell cycle, such as Polo-like kinase [70], [71]. In comparison to the bloodstream form, few alterations to cell cycle progression were detected following CYC9 depletion in procyclic *T. brucei*, and thus, if CYC9 does regulate gene expression, it may regulate expression of different genes in this life cycle stage.

Different phenotypes were observed following CYC9 and CRK12 depletion in bloodstream stage *T. brucei,* which was intriguing given that they form a complex. This may indicate that CYC9 and/or CRK12 interact with additional partners to perform distinct functions, which would be differentially affected by the individual RNAi knockdowns. Alternatively, it is possible that CYC9 and/or CRK12 do indeed play roles in both cytokinesis and endocytosis, but functional redundancy within these pathways may mean that a potential endocytosis function for CYC9 or a potential cytokinesis function for CRK12 is provided by another cyclin or CDK, respectively, in their absence. Additionally, the threshold level of CYC9 or CRK12 required for each of these functions may be different, so that depletion of CYC9 or CRK12 may be sufficient to disrupt cytokinesis or endocytosis, but not both.

Our functional characterisation of CYC9 provides additional evidence that cell cycle regulation varies considerably during the life cycle of *T. brucei*, while our analysis of CRK12 demonstrates for the first time that trypanosomatid CRK functions are not limited to cell cycle regulation. Additionally, our work genetically validates a novel CRK:cyclin complex as a potential drug target in this devastating human and animal pathogen.

## Supporting Information

Figure S1 **Phylogenetic analysis of CYC9 and CRK12.** A: Phylogenetic analysis of CYC9. The cyclin domains of CYC9 and other selected kinetoplastid, human (*H. sapiens*), *Drosophila* (*D. melanogaster*) and yeast (*S. pombe*) cyclins were aligned and bootstrapped as described in the Materials and Methods. *T. brucei* cyclins are highlighted in bold font, transcriptional cyclins are in red font, mitotic cyclins in blue font and stress response cyclins in green font. The CYC9 kinetoplastid cluster is shaded in red. B: Phylogenetic analysis of CRK12. The kinase domains of CRK12 and other selected kinetoplastid, human, *Drosophila* and worm (*C. elegans*) CDKs were aligned and bootstrapped as described in the Materials and Methods. *T. brucei* CRKs are highlighted in bold font, the CRK12 kinetoplastid cluster is shaded in red and the PITSLRE kinases clade is shaded in blue.
(PDF)

Figure S2 **CRK12 and CYC9 interact in a yeast two hybrid assay.** A: β-galactosidase assay for transcription of *LacZ* reporter gene. (a): L40 pHybLex/Zeo pYESTrp; (b): L40 pGL1277 (LexA:CRK12) pYESTrp; (c): L40 pHybLex/Zeo pGL932 (B42:CYC9); (d): L40 pGL1277 pGL932. B. Histidine prototrophy assay. Colonies of yeast strain L40 expressing LexA:Fos/B42:Jun (positive control), LexA:Lamin/B42:Jun (negative control) and LexA:CRK12/B42:CYC9 (two independent transformants) were suspended in PBS, diluted as indicated and spotted onto minimal medium plates containing (+) or lacking (-) histidine (His).
(PDF)

Figure S3 **CYC9:TAP interacts with ty:CRK12 in bloodstream form** ***T. brucei*** **.** A. Immunoprecipitation (IP) of ty:CRK12. Cell lysates co-expressing CYC9:TAP (from the endogenous locus) and ty:CRK12 (under tetracycline inducible control) were incubated with either anti-TY (left panel) or anti-rabbit IgG beads (right panel) before the beads were washed and proteins remaining eluted from the beads. Samples of the input (In), flow through (FT), washes 1 and 3 (W1 and W3) and the elution (E) were analysed by Western blotting with anti-PAP, anti-TY and anti-EF1-α (specificity control) as indicated. D: Immunofluorescence analysis of bloodstream cell line expressing CYC9:TAP. Top right: DIC; top left: DAPI stain for DNA; bottom left: anti-protein A (to detect CYC9:TAP); bottom right: DAPI/anti-protein A merge. Scale bar: 5 µm.
(PDF)

Figure S4 **PCR analysis of putative** ***CYC9*** **knockout cell lines.** Five putative double knockout clonal procyclic cell lines (A) and one putative double knockout clonal bloodstream cell line (B) (each resistant to two different selective drugs) were analysed by PCR alongside appropriate control cells lines (see keys in each part of figure). PCR primers were designed to test correct integration of the 5′ and 3′ flanks of the drug resistance markers used as well as presence of the drug resistance marker ORF, and for the presence of an intact copy of the *CYC9* gene. The expected size of each fragment is indicated. L: 1 kb DNA ladder (see bottom of key for fragment sizes); KO: knockout; *HYG*, *NEO*, *BSD*: resistance genes for hygromycin, neomycin and blasticidin, respectively.
(PDF)

Figure S5 **Depletion of** ***CYC9*** **in procyclic** ***T. brucei*** **does not result in a significant cell cycle defect.** A: DAPI staining of procyclic form *CYC9* RNAi cell lines. Cells were stained with DAPI and the number of nuclei (N) and kinetoplasts (K) per cell quantified at the time points indicated in hours (*n* >300 cells per time point). B: Flow cytometry analysis of procyclic form *CYC9* RNAi. Cells were stained with propidium iodide and analysed by flow cytometry at the time points indicated following induction with tetracycline (tet). The ploidies of the peaks are indicated.
(PDF)

Table S1 **Oligonucleotides used in this study.**
(DOCX)

## References

[1] Satyanarayana A, Kaldis P (2009) Mammalian cell-cycle regulation: several Cdks, numerous cyclins and diverse compensatory mechanisms. Oncogene 28: 2925–2939 onc2009170 [pii];10.1038/onc.2009.170 [doi]. 19561645 10.1038/onc.2009.170

[2] Enserink JM, Kolodner RD (2010) An overview of Cdk1-controlled targets and processes. Cell Div 5: 11 1747-1028-5-11 [pii];10.1186/1747-1028-5-11 [doi]. 20465793 10.1186/1747-1028-5-11 PMC2876151

[3] Nigg EA (1995) Cyclin-dependent protein kinases: Key regulators of the eukaryotic cell cycle. Bioessays 17: 471–480. 7575488 10.1002/bies.950170603

[4] Shiekhattar R, Mermelstein F, Fisher RP, Drapkin R, Dynlacht B, et al (1995) Cdk-activating kinase complex is a component of human transcription factor TFIIH. Nature 374: 283–287. 7533895 10.1038/374283a0

[5] Glover-Cutter K, Larochelle S, Erickson B, Zhang C, Shokat K, et al (2009) TFIIH-associated Cdk7 kinase functions in phosphorylation of C-terminal domain Ser7 residues, promoter-proximal pausing, and termination by RNA polymerase II. Mol Cell Biol 29: 5455–5464 MCB.00637-09 [pii];10.1128/MCB.00637-09 [doi]. 19667075 10.1128/MCB.00637-09 PMC2756882

[6] Choo S, Schroeder S, Ott M (2010) CYCLINg through transcription: posttranslational modifications of P-TEFb regulate transcription elongation. Cell Cycle 9: : 1697–1705. 11346 [pii]. 10.4161/cc.9.9.11346 PMC2956491 20436276

[7] Knuesel MT, Meyer KD, Bernecky C, Taatjes DJ (2009) The human CDK8 subcomplex is a molecular switch that controls Mediator coactivator function. Genes Dev 23: 439–451 23/4/439 [pii];10.1101/gad.1767009 [doi]. 19240132 10.1101/gad.1767009 PMC2648653

[8] Yang Z, Geng J, Yen WL, Wang K, Klionsky DJ (2010) Positive or negative roles of different cyclin-dependent kinase Pho85-cyclin complexes orchestrate induction of autophagy in *Saccharomyces cerevisiae* . Mol Cell 38: 250–264 S1097-2765(10)00250-9 [pii];10.1016/j.molcel.2010.02.033 [doi]. 20417603 10.1016/j.molcel.2010.02.033 PMC2861662

[9] Hisanaga SI, Endo R (2010) Regulation and role of cyclin-dependent kinase activity in neuronal survival and death. J Neurochem 115: 1309–1321 10.1111/j.1471-4159.2010.07050.x [doi]. 21044075 10.1111/j.1471-4159.2010.07050.x

[10] Lenburg ME, Oshea EK (1996) Signaling phosphate starvation. Trends in Biochemical Sciences 21: 383–387. 8918192

[11] Nishizawa M, Tanigawa M, Hayashi M, Maeda T, Yazaki Y, et al (2010) Pho85 kinase, a cyclin-dependent kinase, regulates nuclear accumulation of the Rim101 transcription factor in the stress response of *Saccharomyces cerevisiae* . Eukaryot Cell 9: 943–951 EC.00247-09 [pii];10.1128/EC.00247-09 [doi]. 20382759 10.1128/EC.00247-09 PMC2901641

[12] Morgan DO (1995) Principles of CDK regulation. Nature 374: 131–134. 7877684 10.1038/374131a0

[13] Bloom J, Cross FR (2007) Multiple levels of cyclin specificity in cell-cycle control. Nat Rev Mol Cell Biol 8: 149–160 nrm2105 [pii];10.1038/nrm2105 [doi]. 17245415 10.1038/nrm2105

[14] Barette C, Jariel-Encontre I, Piechaczyk M, Piette J (2001) Human cyclin C protein is stabilized by its associated kinase cdk8, independently of its catalytic activity. Oncogene 20: 551–562. 11313987 10.1038/sj.onc.1204129

[15] Kiernan RE, Emiliani S, Nakayama K, Castro A, Labbe JC, et al (2001) Interaction between cyclin T1 and SCF(SKP2) targets CDK9 for ubiquitination and degradation by the proteasome. Mol Cell Biol 21: 7956–7970 10.1128/MCB.21.23.7956-7970.2001 [doi]. 11689688 10.1128/MCB.21.23.7956-7970.2001 PMC99964

[16] Tassan J-P, Schultz SJ, Bartek J, Nigg EA (1994) Cell cycle analysis of the activity, subcellular localization, and subunit composition of human CAK (CDK-activating kinase). J Cell Biol 127: 467–478. 7929589 10.1083/jcb.127.2.467 PMC2120215

[17] Tsai L-H, Delalle I, Caviness VS Jr, Chae T, Harlow E (1994) p35 is a neural-specific regulatory subunit of cyclin-dependent kinase 5. Nature 371: 419–423. 8090221 10.1038/371419a0

[18] Tang DM, Yeung J, Lee KY, Matsushita M, Matsui H, et al (1995) An isoform of the neuronal cyclin-dependent kinase 5 (Cdk5) activator. J Biol Chem 270: 26897–26903. 7592934 10.1074/jbc.270.45.26897

[19] Tang DM, Chun ACS, Zhang MJ, Wang JH (1997) Cyclin-dependent kinase 5 (CdkB) activation domain of neuronal Cdk5 activator - Evidence of the existence of cyclin fold in neuronal Cdk5a activator. J Biol Chem 272: 12318–12327. 9139676 10.1074/jbc.272.19.12318

[20] Hammarton TC (2007) Cell cycle regulation in *Trypanosoma brucei* . Mol Biochem Parasitol 153: 1–8. 17335918 10.1016/j.molbiopara.2007.01.017 PMC1914216

[21] Naula C, Parsons M, Mottram JC (2005) Protein kinases as drug targets in trypanosomes and *Leishmania* . Biochimica et Biophysica Acta-Proteins and Proteomics 1754: 151–159. 10.1016/j.bbapap.2005.08.018 PMC1452262 16198642

[22] Gourguechon S, Wang CC (2009) CRK9 contributes to regulation of mitosis and cytokinesis in the procyclic form of *Trypanosoma brucei* . BMC Cell Biol 10: 68 1471-2121-10-68 [pii];10.1186/1471-2121-10-68 [doi]. 19772588 10.1186/1471-2121-10-68 PMC2754446

[23] Tu X, Wang CC (2004) The involvement of two cdc2-related kinases (CRKs) in *Trypanosoma brucei* cell-cycle regulation and the distinctive stage-specific phenotypes caused by CRK3 depletion. J Biol Chem 279: 20519–20528. 15010459 10.1074/jbc.M312862200

[24] Badjatia N, Ambrosio DL, Lee JH, Gunzl A (2013) Trypanosome cdc2-related kinase 9 controls spliced leader RNA cap4 methylation and phosphorylation of the RNA polymerase II subunit RPB1. Mol Cell Biol In press. 10.1128/MCB.00156-13 PMC3647971 23478263

[25] Tu X, Wang CC (2005) Coupling of posterior cytoskeletal morphogenesis to the G1/S transition in the *Trypanosoma brucei* cell cycle. Mol Biol Cell 16: 97–105. 15525678 10.1091/mbc.E04-05-0368 PMC539155

[26] Van Hellemond JJ, Neuville P, Schwartz RJ, Matthews KR, Mottram JC (2000) Isolation of *Trypanosoma brucei CYC2* and *CYC3* cyclin genes by rescue of a yeast G_1_ cyclin mutant. Functional characterisation of CYC2. J Biol Chem 275: 8315–8323. 10722661 10.1074/jbc.275.12.8315

[27] Li Z, Wang CC (2003) A PHO80-like cyclin and a B-type cyclin control the cell cycle of the procyclic form of *Trypanosoma brucei* . J Biol Chem 278: 20652–20658. 12665514 10.1074/jbc.M301635200

[28] Hammarton TC, Engstler M, Mottram JC (2004) The *Trypanosoma brucei* cyclin, CYC2, is required for cell cycle progression through G1 phase and maintenance of procyclic form cell morphology. J Biol Chem 279: 24757–24764. 15039435 10.1074/jbc.M401276200

[29] Hammarton TC, Clark J, Douglas F, Boshart M, Mottram JC (2003) Stage-specific differences in cell cycle control in *Trypanosoma brucei* revealed by RNA interference of a mitotic cyclin. J Biol Chem 278: 22877–22886. 12682070 10.1074/jbc.M300813200

[30] Gourguechon S, Savich JM, Wang CC (2007) The multiple roles of cyclin E1 in controlling cell cycle progression and cellular morphology of *Trypanosoma brucei* . J Mol Biol 368: 939–950 S0022-2836(07)00231-8 [pii];10.1016/j.jmb.2007.02.050 [doi]. 17376478 10.1016/j.jmb.2007.02.050 PMC2701699

[31] Thompson JD, Gibson TJ, Plewniak F, Jeanmougin F, Higgins DG (1997) The CLUSTAL_X windows interface: flexible strategies for multiple sequence alignment aided by quality analysis tools. Nucleic Acids Res 25: 4876–4882. gka797 [pii]. 10.1093/nar/25.24.4876 PMC147148 9396791

[32] Bingham J, Sudarsanam S (2000) Visualizing large hierarchical clusters in hyperbolic space. Bioinformatics 16: 660–661. 11038340 10.1093/bioinformatics/16.7.660

[33] Biebinger S, Wirtz E, Lorenz P, Clayton CE (1997) Vectors for inducible expression of toxic gene products in bloodstream and procyclic *Trypanosoma brucei* . Mol Biochem Parasitol 85: 99–112. 9108552 10.1016/s0166-6851(96)02815-0

[34] Wirtz E, Leal S, Ochatt C, Cross GA (1999) A tightly regulated inducible expression system for conditional gene knock-outs and dominant-negative genetics in *Trypanosoma brucei* . Mol Biochem Parasitol 99: 89–101. 10215027 10.1016/s0166-6851(99)00002-x

[35] Alsford S, Horn D (2008) Single-locus targeting constructs for reliable regulated RNAi and transgene expression in *Trypanosoma brucei* . Mol Biochem Parasitol 161: 76–79 S0166-6851(08)00136-9 [pii];10.1016/j.molbiopara.2008.05.006 [doi]. 18588918 10.1016/j.molbiopara.2008.05.006 PMC3828046

[36] Burkard G, Fragoso CM, Roditi I (2007) Highly efficient stable transformation of bloodstream forms of *Trypanosoma brucei* . Mol Biochem Parasitol 153: 220–223. 17408766 10.1016/j.molbiopara.2007.02.008

[37] Rigaut G, Shevchenko A, Rutz B, Wilm M, Mann M, et al (1999) A generic protein purification method for protein complex characterization and proteome exploration. Nat Biotechnol 17: 1030–1032. 10504710 10.1038/13732

[38] Kelly S, Reed J, Kramer S, Ellis S, Webb H, et al (2007) Functional genomics in *Trypanosoma brucei*: a collection of vectors for the expression of tagged proteins from endogenous and ectopic gene loci. Mol Biochem Parasitol 154: 103–109. 17512617 10.1016/j.molbiopara.2007.03.012 PMC2705915

[39] Bastin P, Bagherzadeh A, Matthews KR, Gull K (1996) A novel epitope tag system to study protein targeting and organelle biogenesis in *Trypanosoma brucei* . Mol Biochem Parasitol 77: 235–239. 8813669 10.1016/0166-6851(96)02598-4

[40] Helms MJ, Ambit A, Appleton P, Tetley L, Coombs GH, et al (2006) Bloodstream form *Trypanosoma brucei* depend upon multiple metacaspases associated with RAB11-positive endosomes. J Cell Sci 119: 1105–1117. 16507595 10.1242/jcs.02809

[41] Redmond S, Vadivelu J, Field MC (2003) RNAit: an automated web-based tool for the selection of RNAi targets in *Trypanosoma brucei* . Mol Biochem Parasitol 128: 115–118. 12706807 10.1016/s0166-6851(03)00045-8

[42] LaCount DJ, Bruse S, Hill KL, Donelson JE (2000) Double-stranded RNA interference in *Trypanosoma brucei* using head-to-head promoters. Mol Biochem Parasitol 111: 67–76. 11087917 10.1016/s0166-6851(00)00300-5

[43] Kohler G, Milstein C (1975) Continuous cultures of fused cells secreting antibody of predefined specificity. Nature 256: 495–497. 1172191 10.1038/256495a0

[44] Ma JT, Benz C, Grimaldi R, Stockdale C, Wyatt P, et al (2010) Nuclear DBF-2-related Kinases are essential regulators of cytokinesis in bloodstream stage Trypanosoma brucei. J Biol Chem 285: 15356–15368. 20231285 10.1074/jbc.M109.074591 PMC2865264

[45] Hammarton TC, Ford JR, Mottram JC (2000) *Trypanosoma brucei* CYC1 does not have characteristics of a mitotic cyclin. Mol Biochem Parasitol 111: 229–233. 11087934 10.1016/s0166-6851(00)00308-x

[46] Munday JC, McLuskey K, Brown E, Coombs GH, Mottram JC (2010) Oligopeptidase B deficient mutants of *Leishmania major*. Mol Biochem Parasitol. S0166-6851(10)00234-3 [pii];10.1016/j.molbiopara.2010.09.003 [doi]. 10.1016/j.molbiopara.2010.09.003 PMC3130898 20883728

[47] Mottram JC, Grant KM (1996) *Leishmania mexicana* p12^cks1^, a functional homologue of fission yeast p13^suc1^, associates with a stage-regulated histone H1 kinase. Biochem J 316: 833–839. 8670159 10.1042/bj3160833 PMC1217425

[48] Nolan DP, Jackson DG, Biggs MJ, Brabazon ED, Pays A, et al (2000) Characterization of a novel alanine-rich protein located in surface microdomains in *Trypanosoma brucei* . J Biol Chem 275: 4072–4080. 10660566 10.1074/jbc.275.6.4072

[49] Morgan GW, Allen CL, Jeffries TR, Hollinshead M, Field MC (2001) Developmental and morphological regulation of clathrin-mediated endocytosis in *Trypanosoma brucei* . J Cell Sci 114: 2605–2615. 11683388 10.1242/jcs.114.14.2605

[50] Field MC, Allen CL, Dhir V, Goulding D, Hall BS, et al (2004) New approaches to the microscopic imaging of *Trypanosoma brucei* . Microsc Microanal 10: 621–636 S1431927604040942 [pii];10.1017/S1431927604040942 [doi]. 15525435 10.1017/S1431927604040942

[51] May SF, Peacock L, Almeida Costa CI, Gibson WC, Tetley L, et al. (2012) The *Trypanosoma brucei* AIR9-like protein is cytoskeleton-associated and is required for nucleus positioning and accurate cleavage furrow placement. Mol Microbiol. 10.1111/j.1365-2958.2012.08008.x [doi]. 10.1111/j.1365-2958.2012.08008.x PMC3488599 22329999

[52] Gopinathan L, Ratnacaram CK, Kaldis P (2011) Established and novel Cdk/cyclin complexes regulating the cell cycle and development. Results Probl Cell Differ 53: 365–389 10.1007/978-3-642-19065-0_16 [doi]. 21630153 10.1007/978-3-642-19065-0_16

[53] Allen CL, Goulding D, Field MC (2003) Clathrin-mediated endocytosis is essential in *Trypanosoma brucei* . EMBO J 22: 4991–5002. 14517238 10.1093/emboj/cdg481 PMC204465

[54] Steverding D, Stierhof YD, Fuchs H, Tauber R, Overath P (1995) Transferrin-binding protein complex is the receptor for transferrin uptake in *Trypanosoma brucei* . J Cell Biol 131: 1173–1182. 8522581 10.1083/jcb.131.5.1173 PMC2120630

[55] Mackey ZB, Koupparis K, Nishino M, McKerrow JH (2011) High-throughput analysis of an RNAi library identifies novel kinase targets in *Trypanosoma brucei* . Chem Biol Drug Des 78: 454–463 10.1111/j.1747-0285.2011.01156.x [doi]. 21668652 10.1111/j.1747-0285.2011.01156.x PMC3166884

[56] Langreth SG, Balber AE (1975) Protein uptake and digestion in bloodstream and culture forms of *Trypanosoma brucei* . J Protozool 22: 40–53. 1117436 10.1111/j.1550-7408.1975.tb00943.x

[57] Silverman JS, Schwartz KJ, Hajduk SL, Bangs JD (2011) Late endosomal Rab7 regulates lysosomal trafficking of endocytic but not biosynthetic cargo in *Trypanosoma brucei* . Mol Microbiol 82: 664–678 10.1111/j.1365-2958.2011.07842.x [doi]. 21923766 10.1111/j.1365-2958.2011.07842.x PMC4324464

[58] Hu D, Mayeda A, Trembley JH, Lahti JM, Kidd VJ (2003) CDK11 complexes promote pre-mRNA splicing. J Biol Chem 278: 8623–8629 10.1074/jbc.M210057200 [doi];M210057200 [pii]. 12501247 10.1074/jbc.M210057200

[59] Cornelis S, Bruynooghe Y, Denecker G, Van HS, Tinton S, et al. (2000) Identification and characterization of a novel cell cycle-regulated internal ribosome entry site. Mol Cell 5:: 597–605. S1097-2765(00)80239-7 [pii]. 10.1016/s1097-2765(00)80239-7 10882096

[60] Lahti JM, Xiang J, Kidd VJ (1995) The PITSLRE protein kinase family. Prog Cell Cycle Res 1: 329–338. 9552375 10.1007/978-1-4615-1809-9_27

[61] Trembley JH, Hu D, Hsu LC, Yeung CY, Slaughter C, et al (2002) PITSLRE p110 protein kinases associate with transcription complexes and affect their activity. J Biol Chem 277: 2589–2596 10.1074/jbc.M109755200 [doi];M109755200 [pii]. 11709559 10.1074/jbc.M109755200

[62] Zong H, Chi Y, Wang Y, Yang Y, Zhang L, et al (2007) Cyclin D3/CDK11p58 complex is involved in the repression of androgen receptor. Mol Cell Biol 27: 7125–7142 MCB.01753-06 [pii];10.1128/MCB.01753-06 [doi]. 17698582 10.1128/MCB.01753-06 PMC2168904

[63] Zong H, Li Z, Liu L, Hong Y, Yun X, et al (2005) Cyclin-dependent kinase 11(p58) interacts with HBO1 and enhances its histone acetyltransferase activity. FEBS Lett 579: 3579–3588 S0014-5793(05)00643-5 [pii];10.1016/j.febslet.2005.05.039 [doi]. 15963510 10.1016/j.febslet.2005.05.039

[64] Hu D, Valentine M, Kidd VJ, Lahti JM (2007) CDK11(p58) is required for the maintenance of sister chromatid cohesion. J Cell Sci 120: 2424–2434 120/14/2424 [pii];10.1242/jcs.007963 [doi]. 17606997 10.1242/jcs.007963

[65] Petretti C, Savoian M, Montembault E, Glover DM, Prigent C, et al (2006) The PITSLRE/CDK11p58 protein kinase promotes centrosome maturation and bipolar spindle formation. EMBO Rep 7: 418–424. 16462731 10.1038/sj.embor.7400639 PMC1456919

[66] Xiang J, Lahti JM, Kidd VJ (1994) 2-Aminopurine overrides a late telophase delay created by ectopic expression of the PITSLRE beta 1 protein kinase. Biochem Biophys Res Commun 199: : 1167–1173. S0006291X84713532 [pii]. 10.1006/bbrc.1994.1353 8147857

[67] Wilker EW, van Vugt MA, Artim SA, Huang PH, Petersen CP, et al (2007) 14-3-3sigma controls mitotic translation to facilitate cytokinesis. Nature 446: 329–332 nature05584 [pii];10.1038/nature05584 [doi]. 17361185 10.1038/nature05584

[68] Barna M, Pusic A, Zollo O, Costa M, Kondrashov N, et al (2008) Suppression of Myc oncogenic activity by ribosomal protein haploinsufficiency. Nature 456: 971–975 nature07449 [pii];10.1038/nature07449 [doi]. 19011615 10.1038/nature07449 PMC2880952

[69] Gregory SL, Shandala T, O'Keefe L, Jones L, Murray MJ, et al. (2007) A Drosophila overexpression screen for modifiers of Rho signalling in cytokinesis. Fly (Austin ) 1: : 13–22. 3806 [pii]. 10.4161/fly.3806 18690061

[70] Hammarton TC, Kramer S, Tetley L, Boshart M, Mottram JC (2007) *Trypanosoma brucei* Polo-like kinase is essential for basal body duplication, kDNA segregation and cytokinesis. Mol Microbiol 65: 1229–1248. 17662039 10.1111/j.1365-2958.2007.05866.x PMC2169650

[71] Umeyama T, Wang CC (2008) Polo-like kinase is expressed in S/G(2)/M phase and associated with the flagellum attachment zone in both procyclic and bloodstream forms of *Trypanosoma brucei* . Eukaryotic Cell 7: 1582–1590. 18621923 10.1128/EC.00150-08 PMC2547065

