# Supplementary material for: Identification and Functional Characterisation of CRK12:CYC9, a Novel Cyclin-Dependent Kinase (CDK)-Cyclin Complex in Trypanosoma brucei
Source: PLoS One. 2013 Jun 21;8(6):e67327. doi: 10.1371/journal.pone.0067327 (PMC3689728; doi:10.1371/journal.pone.0067327)
Supplement: Figure S1 — Phylogenetic analysis of CYC9 and CRK12. A: Phylogenetic analysis of CYC9. The cyclin domains of CYC9 and other selected kinetoplastid, human (H. sapiens), Drosophila (D. melanogaster) and yeast (S. pombe) cyclins were aligned and bootstrapped as described in the Materials and Methods. T. brucei cyclins are highlighted in bold font, transcriptional cyclins are in red font, mitotic cyclins in blue font and stress response cyclins in green font. The CYC9 kinetoplastid cluster is shaded in red. B: Phylogenetic analysis of CRK12. The kinase domains of CRK12 and other selected kinetoplastid, human, Drosophila and worm (C. elegans) CDKs were aligned and bootstrapped as described in the Materials and Methods. T. brucei CRKs are highlighted in bold font, the CRK12 kinetoplastid cluster is shaded in red and the PITSLRE kinases clade is shaded in blue. (PDF) [file pone.0067327.s001.pdf]

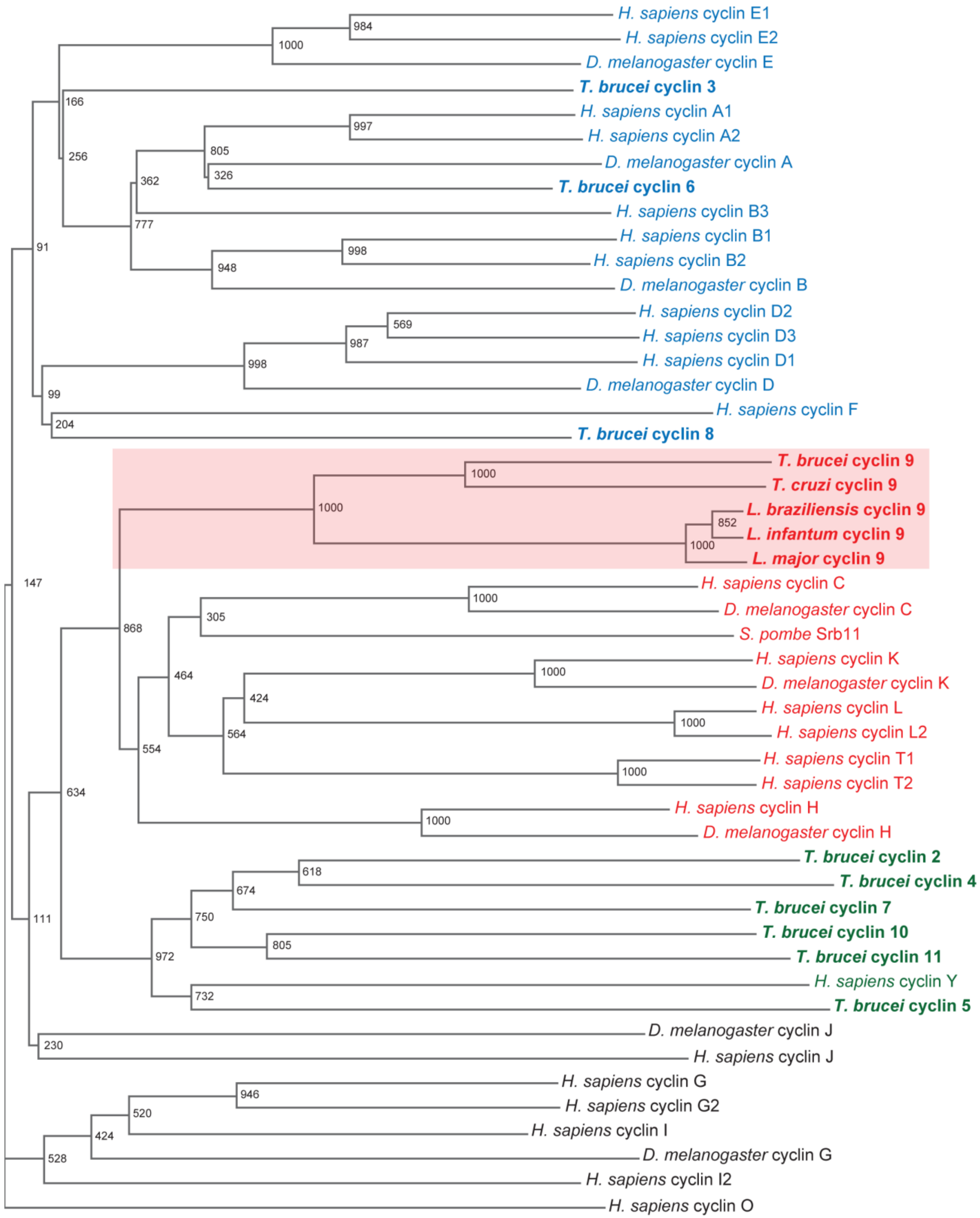
H. sapiens cyclin E1
H. sapiens cyclin E2
D. melanogaster cyclin E
T. brucei cyclin 3
H. sapiens cyclin A1
H. sapiens cyclin A2
D. melanogaster cyclin A
T. brucei cyclin 6
H. sapiens cyclin B3
H. sapiens cyclin B1
H. sapiens cyclin B2
D. melanogaster cyclin B
H. sapiens cyclin D2
H. sapiens cyclin D3
H. sapiens cyclin D1
D. melanogaster cyclin D
H. sapiens cyclin F
T. brucei cyclin 8
T. brucei cyclin 9
T. cruzi cyclin 9
L. braziliensis cyclin 9
L. infantum cyclin 9
L. major cyclin 9
H. sapiens cyclin C
D. melanogaster cyclin C
S. pombe Srb11
H. sapiens cyclin K
D. melanogaster cyclin K
H. sapiens cyclin L
H. sapiens cyclin L2
H. sapiens cyclin T1
H. sapiens cyclin T2
H. sapiens cyclin H
D. melanogaster cyclin H
T. brucei cyclin 2
T. brucei cyclin 4
T. brucei cyclin 7
T. brucei cyclin 10
T. brucei cyclin 11
H. sapiens cyclin Y
T. brucei cyclin 5
D. melanogaster cyclin J
H. sapiens cyclin J
H. sapiens cyclin G
H. sapiens cyclin G2
H. sapiens cyclin I
D. melanogaster cyclin G
H. sapiens cyclin I2
H. sapiens cyclin O
984
1000
166
256
997
805
326
362
777
91
998
948
569
987
998
99
204
1000
1000
852
1000
147
1000
305
868
464
1000
424
1000
564
554
1000
1000
634
618
674
750
805
972
732
111
230
946
520
424
528

Figure S1A

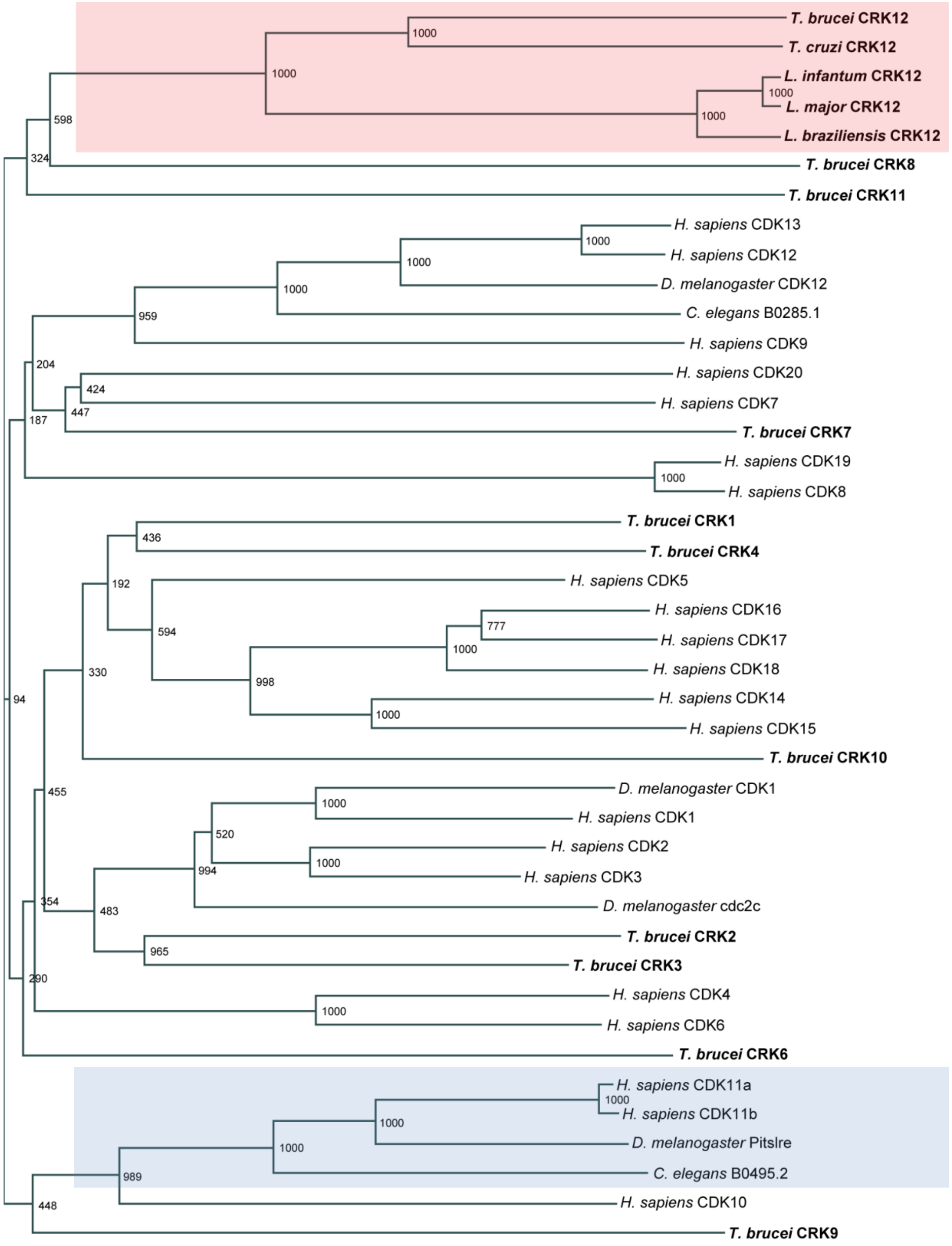
T. brucei CRK12
T. cruzi CRK12
L. infantum CRK12
L. major CRK12
L. braziliensis CRK12
T. brucei CRK8
T. brucei CRK11
H. sapiens CDK13
H. sapiens CDK12
D. melanogaster CDK12
C. elegans B0285.1
H. sapiens CDK9
H. sapiens CDK20
H. sapiens CDK7
T. brucei CRK7
H. sapiens CDK19
H. sapiens CDK8
T. brucei CRK1
T. brucei CRK4
H. sapiens CDK5
H. sapiens CDK16
H. sapiens CDK17
H. sapiens CDK18
H. sapiens CDK14
H. sapiens CDK15
T. brucei CRK10
D. melanogaster CDK1
H. sapiens CDK1
H. sapiens CDK2
H. sapiens CDK3
D. melanogaster cdc2c
T. brucei CRK2
T. brucei CRK3
H. sapiens CDK4
H. sapiens CDK6
T. brucei CRK6
H. sapiens CDK11a
H. sapiens CDK11b
D. melanogaster Pitslre
C. elegans B0495.2
H. sapiens CDK10
T. brucei CRK9
1000
598
324
204
959
424
447
187
436
192
594
777
998
330
94
455
520
994
354
483
965
290
989
448

Figure S1B

Figure S1: Phylogenetic analysis of CYC9 and CRK12. A: Phylogenetic analysis of CYC9. The cyclin domains of CYC9 and other selected kinetoplastid, human (*H. sapiens*), *Drosophila* (*D. melanogaster*) and yeast (*S. pombe*) cyclins were aligned and bootstrapped as described in the Materials and Methods. *T. brucei* cyclins are highlighted in bold font, transcriptional cyclins are in red font, mitotic cyclins in blue font and stress response cyclins in green font. The CYC9 kinetoplastid cluster is shaded in red. B: Phylogenetic analysis of CRK12. The kinase domains of CRK12 and other selected kinetoplastid, human, *Drosophila* and worm (*C. elegans*) CDKs were aligned and bootstrapped as described in the Materials and Methods. *T. brucei* CRKs are highlighted in bold font, the CRK12 kinetoplastid cluster is shaded in red and the PITSLRE kinases clade is shaded in blue.
