# Supplementary material for: Identification and Functional Characterisation of CRK12:CYC9, a Novel Cyclin-Dependent Kinase (CDK)-Cyclin Complex in Trypanosoma brucei
Source: PLoS One. 2013 Jun 21;8(6):e67327. doi: 10.1371/journal.pone.0067327 (PMC3689728; doi:10.1371/journal.pone.0067327)
Supplement: Figure S2 — CRK12 and CYC9 interact in a yeast two hybrid assay. A: β-galactosidase assay for transcription of LacZ reporter gene. (a): L40 pHybLex/Zeo pYESTrp; (b): L40 pGL1277 (LexA:CRK12) pYESTrp; (c): L40 pHybLex/Zeo pGL932 (B42:CYC9); (d): L40 pGL1277 pGL932. B. Histidine prototrophy assay. Colonies of yeast strain L40 expressing LexA:Fos/B42:Jun (positive control), LexA:Lamin/B42:Jun (negative control) and LexA:CRK12/B42:CYC9 (two independent transformants) were suspended in PBS, diluted as indicated and spotted onto minimal medium plates containing (+) or lacking (-) histidine (His). (PDF) [file pone.0067327.s002.pdf]

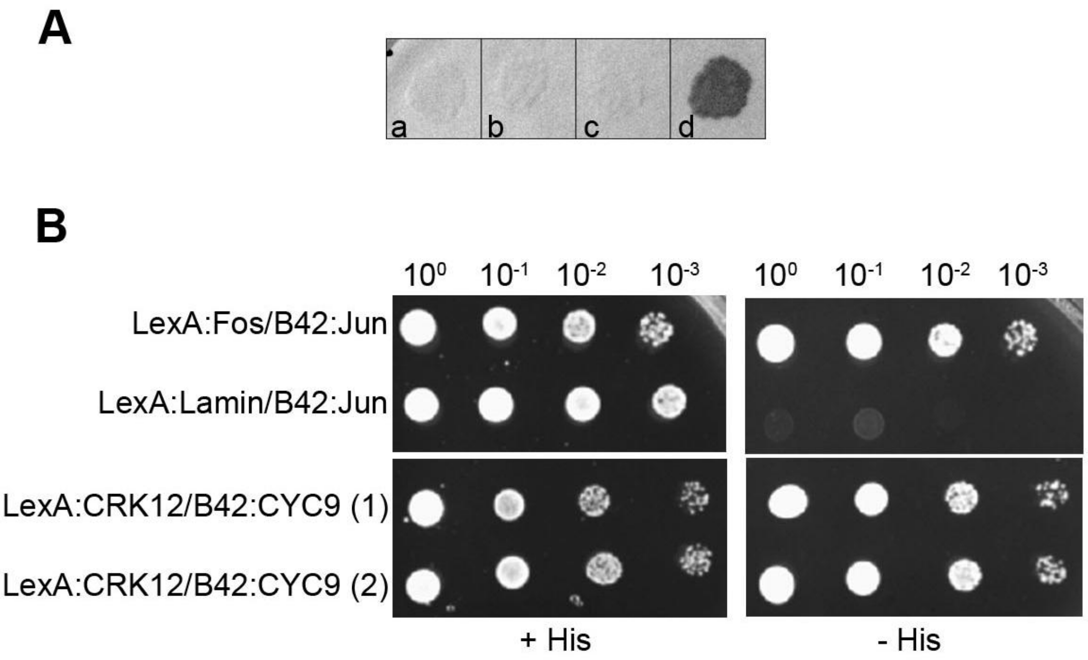

Figure S2. CRK12 and CYC9 interact in a yeast two hybrid assay. A: β-galactosidase assay for transcription of *LacZ* reporter gene. (a): L40 pHybLex/Zeo pYESTrp; (b): L40 pGL1277 (LexA:CRK12) pYESTrp; (c): L40 pHybLex/Zeo pGL932 (B42:CYC9); (d): L40 pGL1277 pGL932. B. Histidine prototrophy assay. Colonies of yeast strain L40 expressing LexA:Fos/B42:Jun (positive control), LexA:Lamin/B42:Jun (negative control) and LexA:CRK12/B42:CYC9 (two independent transformants) were suspended in PBS, diluted as indicated and spotted onto minimal medium plates containing (+) or lacking (-) histidine (His).

Figure S2
