# Supplementary material for: Identification and Functional Characterisation of CRK12:CYC9, a Novel Cyclin-Dependent Kinase (CDK)-Cyclin Complex in Trypanosoma brucei
Source: PLoS One. 2013 Jun 21;8(6):e67327. doi: 10.1371/journal.pone.0067327 (PMC3689728; doi:10.1371/journal.pone.0067327)
Supplement: Figure S3 — CYC9:TAP interacts with ty:CRK12 in bloodstream form T. brucei . A. Immunoprecipitation (IP) of ty:CRK12. Cell lysates co-expressing CYC9:TAP (from the endogenous locus) and ty:CRK12 (under tetracycline inducible control) were incubated with either anti-TY (left panel) or anti-rabbit IgG beads (right panel) before the beads were washed and proteins remaining eluted from the beads. Samples of the input (In), flow through (FT), washes 1 and 3 (W1 and W3) and the elution (E) were analysed by Western blotting with anti-PAP, anti-TY and anti-EF1-α (specificity control) as indicated. D: Immunofluorescence analysis of bloodstream cell line expressing CYC9:TAP. Top right: DIC; top left: DAPI stain for DNA; bottom left: anti-protein A (to detect CYC9:TAP); bottom right: DAPI/anti-protein A merge. Scale bar: 5 µm. (PDF) [file pone.0067327.s003.pdf]

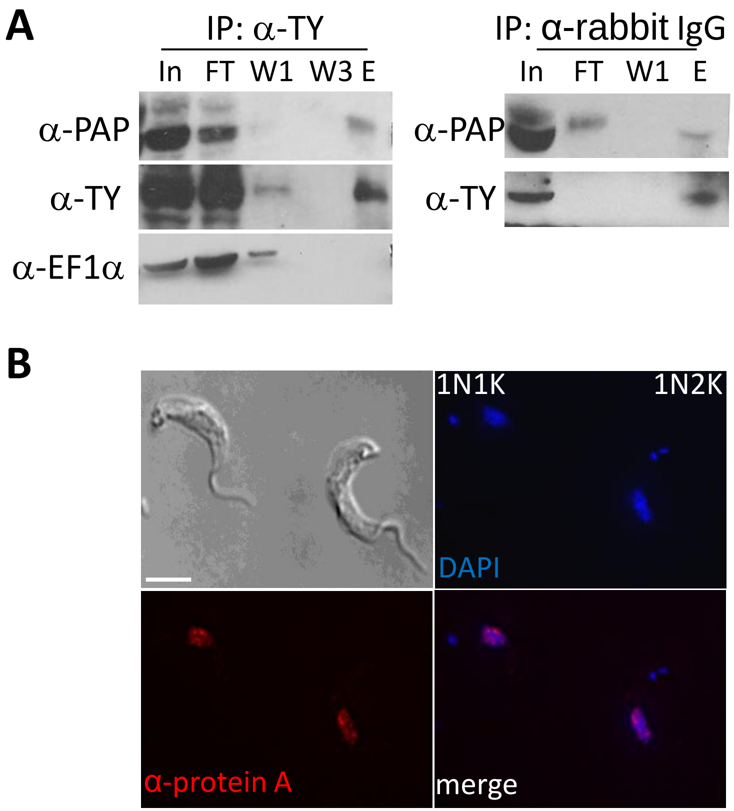

Figure S3: CYC9:TAP interacts with ty:CRK12 in bloodstream form *T. brucei*. A. Immunoprecipitation (IP) of ty:CRK12. Cell lysates co-expressing CYC9:TAP (from the endogenous locus) and ty:CRK12 (under tetracycline inducible control) were incubated with either anti-TY (left panel) or anti-rabbit IgG beads (right panel) before the beads were washed and proteins remaining eluted from the beads. Samples of the input (In), flow through (FT), washes 1 and 3 (W1 and W3) and the elution (E) were analysed by Western blotting with anti-PAP, anti-TY and anti-EF1-α (specificity control) as indicated. D: Immunofluorescence analysis of bloodstream cell line expressing CYC9:TAP. Top right: DIC; top left: DAPI stain for DNA; bottom left: anti-protein A (to detect CYC9:TAP); bottom right: DAPI/anti-protein A merge. Scale bar: 5 µm.

Figure S3
