# Supplementary material for: Identification and Functional Characterisation of CRK12:CYC9, a Novel Cyclin-Dependent Kinase (CDK)-Cyclin Complex in Trypanosoma brucei
Source: PLoS One. 2013 Jun 21;8(6):e67327. doi: 10.1371/journal.pone.0067327 (PMC3689728; doi:10.1371/journal.pone.0067327)
Supplement: Figure S4 — PCR analysis of putative CYC9 knockout cell lines. Five putative double knockout clonal procyclic cell lines (A) and one putative double knockout clonal bloodstream cell line (B) (each resistant to two different selective drugs) were analysed by PCR alongside appropriate control cells lines (see keys in each part of figure). PCR primers were designed to test correct integration of the 5′ and 3′ flanks of the drug resistance markers used as well as presence of the drug resistance marker ORF, and for the presence of an intact copy of the CYC9 gene. The expected size of each fragment is indicated. L: 1 kb DNA ladder (see bottom of key for fragment sizes); KO: knockout; HYG, NEO, BSD: resistance genes for hygromycin, neomycin and blasticidin, respectively. (PDF) [file pone.0067327.s004.pdf]

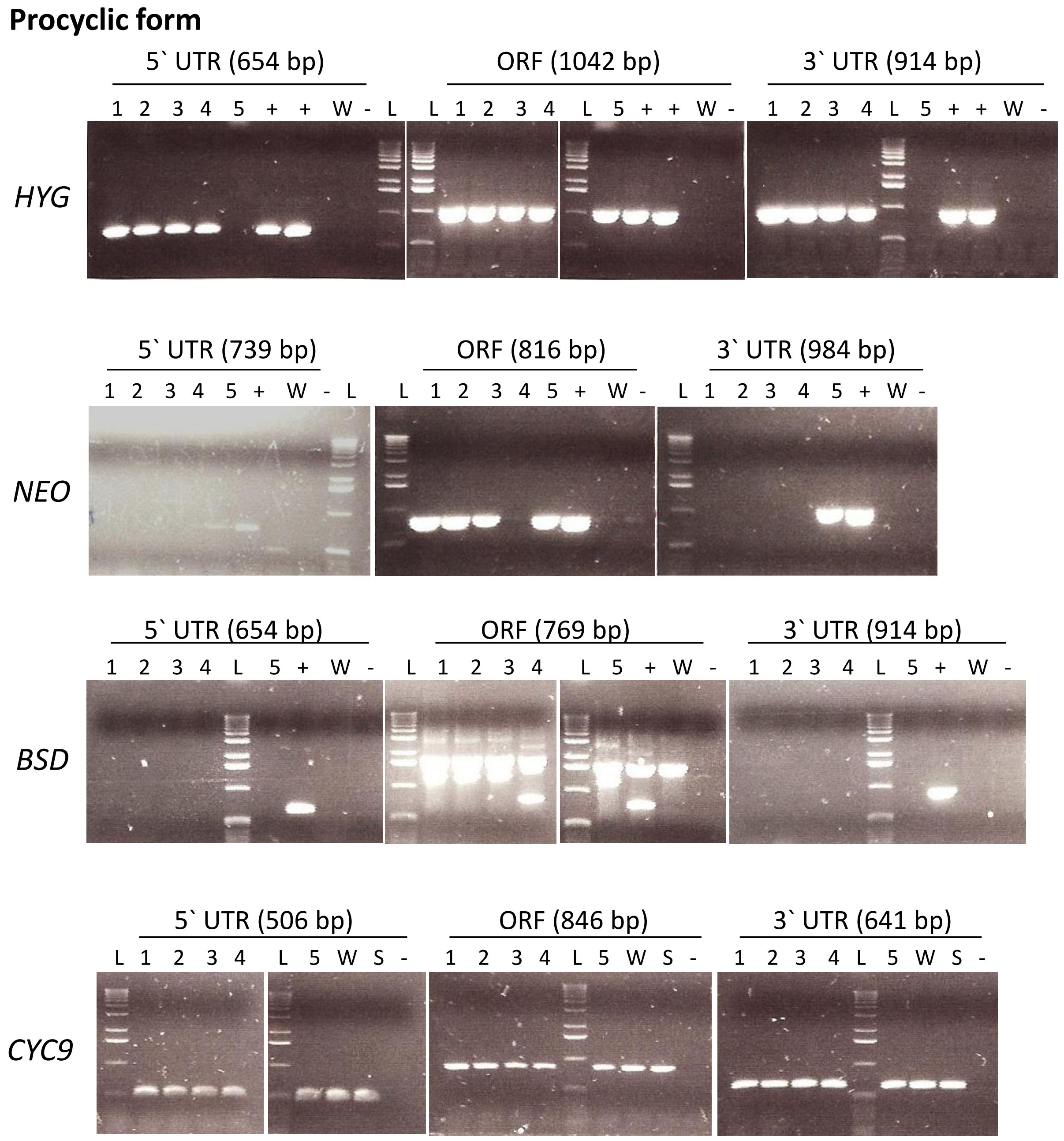

Key

Lane	Cell line	1st allele KO	2nd allele KO
1	Clone 2E9	*HYG*	*NEO*
2	Clone 2A10	*HYG*	*NEO*
3	Clone 3C7	*HYG*	*NEO*
4	Clone 2C4	*HYG*	*BSD*
5	Clone 1E1	*NEO*	*HYG*
+	positive control	Single allele *CYC9* KO with appropriate resistance marker	
W	427 wildtype	n/a	n/a
-	No DNA control	n/a	n/a
S	Single allele *CYC9* KO	HYG	n/a

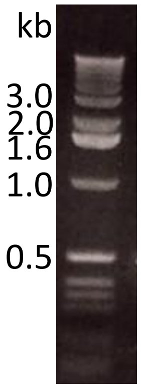

Figure S4A

Bloodstream form

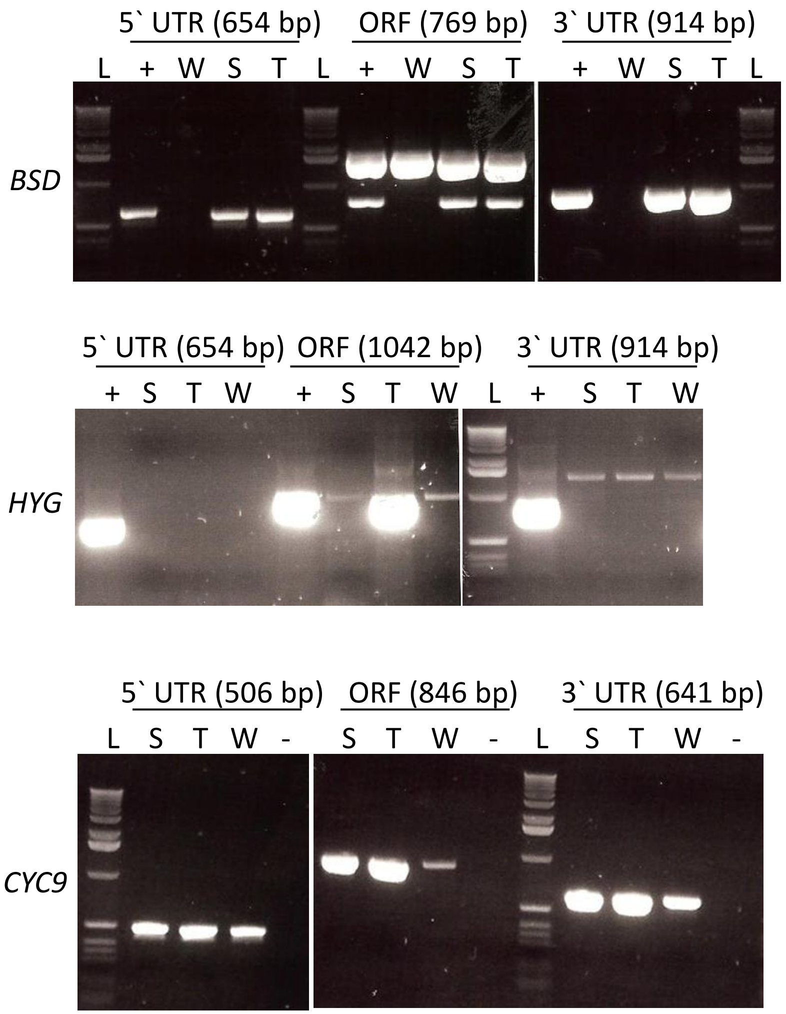

Key

Lane	Cell line	1st allele KO	2nd allele KO
+	positive control	Single allele *CYC9* KO (PCF) with appropriate resistance marker	
W	427 wildtype	n/a	n/a
S	Single allele *CYC9* KO	*BSD*	n/a
T	Clone 1C6	*BSD*	*HYG*
-	No DNA control	n/a	n/a

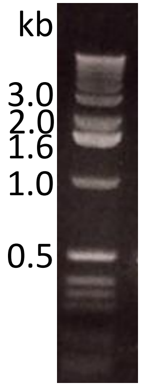

Figure S4B

Figure S4: PCR analysis of putative *CYC9* knockout cell lines. Five putative double knockout clonal procyclic cell lines (A) and one putative double knockout clonal bloodstream cell line (B) (each resistant to two different selective drugs) were analysed by PCR alongside appropriate control cells lines (see keys in each part of figure). PCR primers were designed to test correct integration of the 5` and 3` flanks of the drug resistance markers used as well as presence of the drug resistance marker ORF, and for the presence of an intact copy of the *CYC9* gene. The expected size of each fragment is indicated. L: 1 kb DNA ladder (see bottom of key for fragment sizes); KO: knockout; *HYG*, *NEO*, *BSD*: resistance genes for hygromycin, neomycin and blasticidin, respectively.
