# Supplementary material for: Identification and Functional Characterisation of CRK12:CYC9, a Novel Cyclin-Dependent Kinase (CDK)-Cyclin Complex in Trypanosoma brucei
Source: PLoS One. 2013 Jun 21;8(6):e67327. doi: 10.1371/journal.pone.0067327 (PMC3689728; doi:10.1371/journal.pone.0067327)
Supplement: Figure S5 — Depletion of CYC9 in procyclic T. brucei does not result in a significant cell cycle defect. A: DAPI staining of procyclic form CYC9 RNAi cell lines. Cells were stained with DAPI and the number of nuclei (N) and kinetoplasts (K) per cell quantified at the time points indicated in hours (n >300 cells per time point). B: Flow cytometry analysis of procyclic form CYC9 RNAi. Cells were stained with propidium iodide and analysed by flow cytometry at the time points indicated following induction with tetracycline (tet). The ploidies of the peaks are indicated. (PDF) [file pone.0067327.s005.pdf]

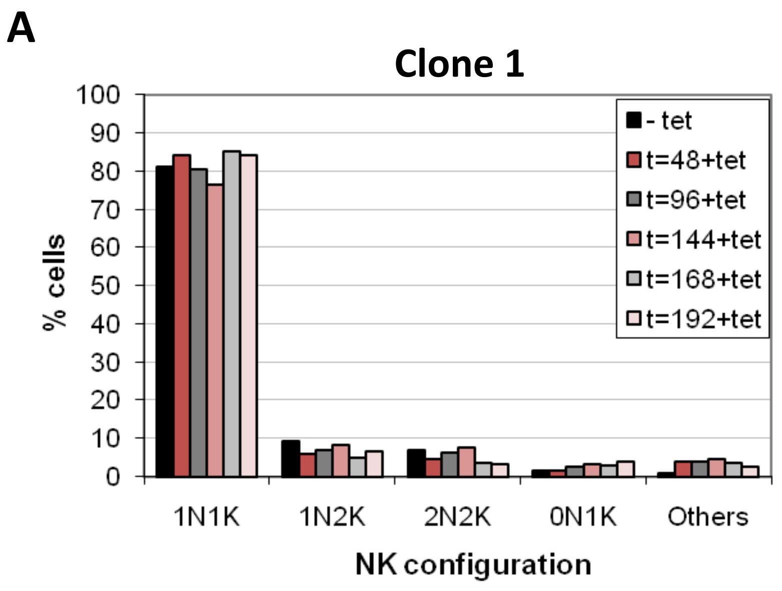
A
Clone 1
% cells
- tet
t=48+tet
t=96+tet
t=144+tet
t=168+tet
t=192+tet
1N1K
1N2K
2N2K
0N1K
Others
NK configuration
Clone 2
% cells
- tet
t=24+tet
t=48+tet
t=72+tet
t=144+tet
t=168+tet
t=216+tet
1N1K
1N2K
2N2K
0N1K
Others
NK configuration

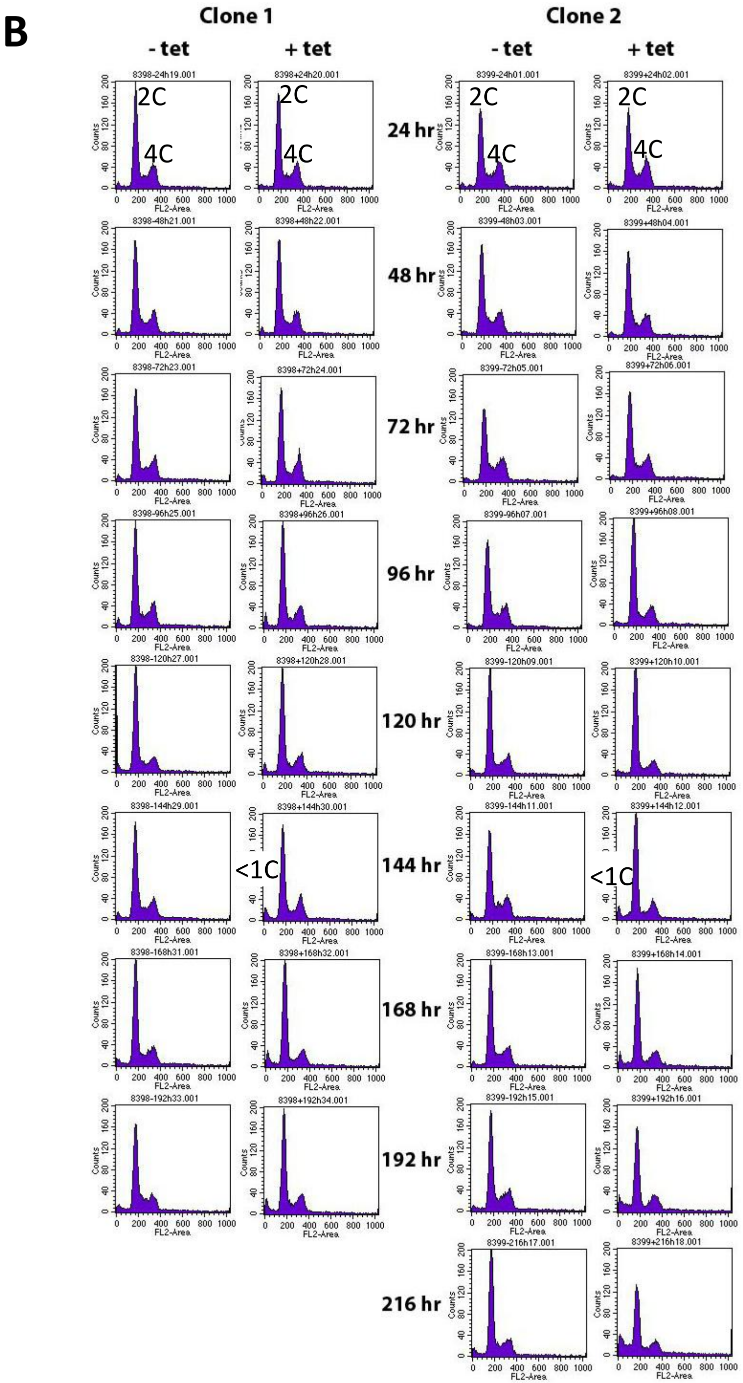
B
Clone 1
Clone 2
- tet
+ tet
- tet
+ tet
2C
4C
<1C
24 hr
48 hr
72 hr
96 hr
120 hr
144 hr
168 hr
192 hr
216 hr
Counts
FL2-Area

Figure S5

Figure S5: Depletion of *CYC9* in procyclic *T. brucei* does not result in a significant cell cycle defect. A: DAPI staining of procyclic form *CYC9* RNAi cell lines. Cells were stained with DAPI and the number of nuclei (N) and kinetoplasts (K) per cell quantified at the time points indicated in hours (*n* >300 cells per time point). B: Flow cytometry analysis of procyclic form *CYC9* RNAi. Cells were stained with propidium iodide and analysed by flow cytometry at the time points indicated following induction with tetracycline (tet). The ploidies of the peaks are indicated.
