# Supplementary material for: Identification and Functional Characterisation of CRK12:CYC9, a Novel Cyclin-Dependent Kinase (CDK)-Cyclin Complex in Trypanosoma brucei
Source: PLoS One. 2013 Jun 21;8(6):e67327. doi: 10.1371/journal.pone.0067327 (PMC3689728; doi:10.1371/journal.pone.0067327)
Supplement: Table S1 — Oligonucleotides used in this study. (DOCX) [file pone.0067327.s006.docx]

Table S1

*Oligonucleotides used in this study*

| **Oligo** | **Details** | **Sequence 5`-3`** |
| --- | --- | --- |
| OL13 | To check correct integration of *HYG* 5`UTR, antisense | GGTGAGTTCAGGCTTTTTCA |
| OL14 | To check correct integration of *HYG* 3`UTR, sense | CGTCCGAGGGCAAAGGAATA |
| OL22 | To check correct integration of *NEO* 5`UTR, antisense | CGATTGTCTGTTGTGCCCAG |
| OL139 | To check for presence of *NEO* ORF, sense | ACTAGTATGGGATCGGCCATTGAACAAG (*Spe* I) |
| OL140 | To check for presence of *NEO* ORF, antisense | GGATCCTCAGAAGAACTCGTCAAGAAG (*Bam H*I) |
| OL536 | To check correct integration of *BSD* 5`UTR, antisense | TTGAGACAAAGGCTTGGCCAT |
| OL537 | To check correct integration of *BSD* 3`UTR, sense | GGTTATGTGTGGGAGGGCTAA |
| OL982 | To check for presence of intact *CYC9* 5`UTR, antisense | GCCTGGATAGCAATGGTATAG |
| OL1144 | To check for presence of *BSD* ORF, sense | AGCAACAGTAGGTACTAGCACC |
| OL1145 | To check for presence of *BSD* ORF, antisense | CTTTCTCCTCTAGTAGGGCTGG |
| OL1163 | *CYC9* ORF for yeast two-hybrid, sense | CAGCAAGCTTATGGCAGGTTTCACCGAG (*Hind* III) |
| OL1164 | *CYC9* ORF for yeast two-hybrid, antisense | GTTGGGATTCTCACTCCACCGATGGTGC (*Bam H*I) |
| OL1307 | To check for presence of *HYG* ORF, sense | GCGAATTCATGAAAAAGCCTGAACTCACCGC (*Eco R*I) |
| OL1308 | To check for presence of *HYG* ORF, antisense | GCGAATTCCTATTCCTTTGCCCTCGGACG (*Eco R*I) |
| OL1360 | To check correct integration of *NEO* 3`UTR, sense | GTGCTTTACGGTATCGCCGC |
| OL1553 | 5` flank *CYC9*, sense | GCGGCCGCAAAAGATATGCTTGCTTTTTCCTTT (*Not* I) |
| OL1554 | 5` flank *CYC9*, antisense | TCTAGAAACGCCACAATCGGACACAAC (*Xba* I) |
| OL1547 | 3` end *CYC9* ORF, sense | CCATGGCTCGAGATTAACGAGTTAGTTTTTGATGGT (*Nco* I, *Xho* I) |
| OL1548 | 3` end *CYC9* ORF, antisense | CCATGGCCTCCACCGATGGTGCACAA (*Nco* I) |
| OL1549 | 3` UTR *CYC9*, sense | GGGCCCTGATGTCTCGTCGACTGATTTG (*Apa* I) |
| OL1550 | 3` UTR *CYC9*, antisense | GGGCCCCTCGAGAAGGAAGTAAAGGACAAACAAAGT (*Apa* I, *Xho* I) |
| OL1600 | *CRK12* ORF for yeast two-hybrid, sense | GTGAGAGCTCATGGGTATGGCAACACGTTC (*Sac* I) |
| OL1601 | *CRK12* ORF for yeast two-hybrid, sense | GTTTGGTACCTTACGCAGCGCTTGGATAAG (*Kpn* I) |
| OL1712 | To check correct integration of 3`UTR, antisense | CTAACGCCCTCTCACCGGG |
| OL1713 | To check correct integration of 5`UTR, sense | TGCCCACAACTTCAAGAAGGA |
| OL1837 | To check for presence of *CYC9* ORF, antisense | GGATCCTCACTCCACCGATGGTGCAC (*Bam H*I) |
| OL1838 | To check for presence of *CYC9* ORF, sense | AAGCTTATGGCAGGTTTCACCGAGACA (*Hind* III) |
| OL1840 | To check for presence of *CYC9* 3`UTR, sense | CGTAAAAGACTTCGTGAACAGG |
| OL2540 | *CYC9* fragment for RNAi, sense | AACCAAGCTTAGGTAGACCCTGTCGGAGGT (*Hind III*) |
| OL2541 | *CYC9* fragment for RNAi, antisense | AATTGGATCCGTCGCGCAGAGATCACATTA (*Bam H*I) |
| OL3387 | *CRK12* fragment for RNAi, sense | GATCCCCGGGCTCGAGTATACATTGGTCGTTGCCGA (*Xma*I, *Xho*I) |
| OL3388 | *CRK12* fragment for RNAi, antisense | CATGGGATCCTCTAGAGAGCACATTGTCAGCCTTGA (*BamH*I, *Xba*I) |
| PR32 | 5` end *CRK12* ORF, sense | GGAGTCTAGAGGTATGGCAACACGTTCGC (*Xba* I) |
| PR33 | 5` end *CRK12* ORF, antisense | CCAACTCGAGCAACCCTCAACGAACGCAAAC (*Xho* I) |
| PR34 | 5` end *CRK12* UTR, sense | GTCCCTCGAGCTGTCCCTCATTGTTAGTG (*Xho* I) |
| PR35 | 5` end *CRK12* UTR, antisense | GTGGGGATCCGGAACAAATCGTCTCGTGACAGTG (*Bam H*I) |
| PR206 | *ty:CRK12*, sense | CGCGAGATCTATGGAGGTCCATACTAACCAAGATCCACTTGACGGTATGGCAACACGTTCGCCTTCGCC (*Bgl* II) |
| PR207 | *CRK12* ORF, antisense | GTTGAGATCTTTACGCAGCGCTTGGATAAGG (*Bgl II*) |
| PR208 | *CRK12* K358M site directed mutagenesis, sense | GGTGAGTATGTTGCGTTGATGCGACTTAAAGTTTTAGAGG |
| PR209 | *CRK12* K358M site directed mutagenesis, antisense | CCTCTAAAACTTTAAGTCGCATCAACGCAACATACTCACC |
| OL2272 | *GPI8*, real time PCR, sense | CGAAGCGCATTTGGATAGC |
| OL2273 | *GPI8*, real time PCR, antisense | AGCGCGTGATGACAGTGAAG |
| OL2314 | *CYC9* real time PCR, sense | GGTGCGGCTGAGCGATAT |
| OL2315 | *CYC9* real time PCR, antisense | CACGCCAAGACTCCATCACA |
| OL3426 | *CRK12* real time PCR, sense | CGCCCGCCTGAGATGTTA |
| OL3427 | *CRK12* real time PCR, antisense | GCAGCCAATAGACCATATATCAACCT |

Restriction sites, where incorporated into the oligo, are underlined.
